# Analytical and simulation studies of driven diffusive system with asymmetric heterogeneous interactions

**DOI:** 10.1038/s41598-018-34579-1

**Published:** 2018-11-02

**Authors:** Yu-Qing Wang, Ji-Xin Wang, Wan-He Li, Chao-Fan Zhou, Bin Jia

**Affiliations:** 1 School of Mechanical Engineering, Hefei University of Technology, Hefei, 230009 China; 2 MOE Key Laboratory for Urban Transportation Complex Systems Theory and Technology, Beijing Jiaotong University, Beijing, 100044 China; 3 School of Physical Sciences, University of Science and Technology of China, Hefei, 230026 China

## Abstract

Totally asymmetric simple exclusion process (namely, TASEP) is one of the most vital driven diffusive systems, which depicts stochastic dynamics of self-driven particles unidirectional updating along one-dimensional discrete lattices controlled by hard-core exclusions. Different with pre-existing results, driven diffusive system composed by multiple TASEPs with asymmetric heterogeneous interactions under two-dimensional periodic boundaries is investigated. By using detailed balance principle, particle configurations are extensively studied to obtain universal laws of characteristic order parameters of such stochastic dynamic system. By performing analytical analyses and Monte-Carlo simulations, local densities are found to be monotone increase with global density and spatially homogeneous to site locations. Oppositely, local currents are found to be non-monotonically increasing against global density and proportional to forward rate. Additionally, by calculating different cases of topologies, changing transition rates are found to have greater effects on particle configurations in adjacent subsystems. By intuitively comparing with pre-existing results, the improvement of our work also shows that introducing and considering totally heterogeneous interactions can improve the total current in such multiple TASEPs and optimize the overall transport of such driven-diffusive system. Our research will be helpful to understand microscopic dynamics and non-equilibrium dynamical behaviors of interacting particle systems.

## Introduction

Driven diffusive system has aroused significant attentions for its importance in non-equilibrium statistical physics, which intuitively reflects profound non-equilibrium dynamic mechanisms and contains deep non-equilibrium characteristics^1–4^. Being one of the most critical kinds of driven diffusive system, totally asymmetric simple exclusion process (namely, TASEP) illustrates motion of self-driven particles along one-dimensional discrete lattices at specific rates^5–7^. Affected by hard-core exclusions, TASEP can well depict complex non-equilibrium dynamical phenomena, such as traffic flow^8,9^, pedestrian flow^10^, quantum dots transport^11^, intercellular transport^12–14^, transcription factors transport^15,16^ etc. Thus, regarded as a paradigm like Ising model, it has attracted considerable attentions for its fundamentality in understanding essential mechanisms like shuffled dynamics^17^, cluster dynamics^18–20^, spontaneous symmetry breaking^21–23^, domain wall theories^24–29^, phase separation^30–32^ etc.

Generally, interactions among subsystems have a great impact on non-equilibrium properties of driven diffusive system. As for one-dimensional TASEP, the system is comprehensively studied by two main methods, namely mean-field approximations^26,33,34^ and exact analyses^35–38^. Additionally, as for multi-lane TASEPs, the global system is composed of several subsystems based on one-dimensional TASEP. In tremendous circumstances, mean-field approximation is often chosen to investigate such multi-lane TASEPs for its simplicity of considering interactions of particles, especially spatial correlation. However, universal evolution laws of characteristic order parameters of such stochastic dynamic systems can hardly be obtained by employing methods with approximations. This is because that these laws should be independent of specific values of parameters, which can be realized by performing strict mathematical derivations without approximation conditions (namely, analytical analyses). Additionally, Ezaki firstly performed the pioneering study about exact analyses of a heterogeneous system composed of multi-lane TASEPs with symmetric lane-changing rates^39^. Here, symmetric lane-changing rates mean that upward and downward rates for each subsystem are equal to each other (namely, the rate ${{\rm{\chi }}}_{i}$)^39^. However, those for any two subsystems are different (namely, ${{\rm{\chi }}}_{i}\ne {{\rm{\chi }}}_{j}$ for ∀*i* ≠ *j*)^39^. Thus, ref.^39^ is the case of partly heterogeneous interactions. Afterwards, authors focused on asymmetric lane-changing behavior under two strong constraint conditions, where two special cases (namely, equal rates for internal and external adjacent lanes) were studied^40^. The heterogeneity of multi-lane TASEPs was also partly considered.

Here, relevant to transport process of driven diffusive systems in real world and motivated by it, we aim to study a two-dimensional driven diffusive system with generalized heterogeneous interactions, which is more capable of depicting real transport phenomena. For instance, as for traffic flow, vehicles can be driven along their own lanes or perform lane-changing behavior at different rates^41–43^. Another case is unidirectional motion of protein motors which can be divided into different movements including directional moving along filaments, detaching from them and diffusing into the surrounding cytoplasm at different rates^44–47^. Thus, above-mentioned phenomena can be modelled by our work, namely multi-channel TASEPs with asymmetric heterogeneous transition rates. Actually, heterogeneity is not fully considered in previous work^39^. Only symmetric heterogeneous interactions were discussed, which led to conclusions relatively lack of universality and not fully reflecting influence of heterogeneity on characteristic order parameters (namely, current, density etc.) of such stochastic system.

Different from pre-existing results, asymmetric heterogeneous interactions are introduced, which fully reflect heterogeneity of subsystems. In details, the interaction between lanes i and ${\rm{i}}-1$ is not equal to that between lanes i and ${\rm{i}}+1$, which means that heterogeneous interactions are asymmetric. Additionally, transition rates for each lane are arbitrary. Thus, we aim at investigating a driven diffusive system with totally heterogeneous interactions. The proposed two-dimensional stochastic system is composed of multiple TASEPs with periodic boundaries. Asymmetric transition rates among adjacent subsystems dominate non-equilibrium dynamic characteristics of the system. The goal of our work is proposing a more universal interacting multi-body particle system more reliable to depict real transport phenomena, constructing such driven diffusive system by employing multiple TASEPs with totally heterogeneous interactions, obtaining analytical solutions and Monte-Carlo simulations to avoid using previous approximations leading to results lack of universal laws, obtaining universal laws of characteristic order parameters by calculating more complex topologies rather than previous four TASEPs^39^ and intuitively present improvements of our research by clearly comparing with pre-existing results though investigating the effect of heterogeneous interactions on overall transport and comparing relationship among total current, global density and scaling rate in three complete kinds of cases (namely, totally heterogeneous, partly heterogeneous and homogeneous) besides in the technical perspective.

In order to perform analytical analyses, detailed balance principle is firstly analyzed. Transitions among complete configurations of particles are fully considered. Then, exact results of the restriction of density weight are obtained. Afterwards, by employing complex analysis and Monte-Carlo simulations, heterogeneous interactions are extensively analyzed by reporting calculations of characteristic order parameters. Additionally, different topologies rather than previous four TASEPs^39^, are calculated to investigate effect of changing transition rate on local density and increment of total current. Finally, impact of heterogeneous interactions on overall transport is studied.

## Results

### Asymmetric heterogeneous model

**Figure 1:**
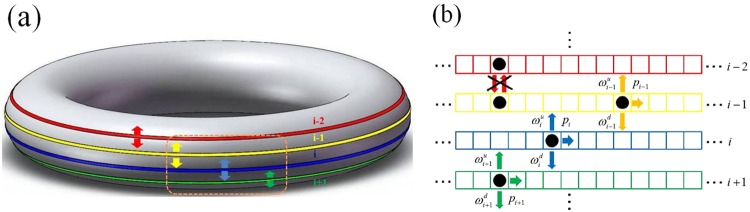
Sketch of model. (**a**) Intuitionistic description. (**b**) Update rules, an enlarged view of orange dotted box. Four adjacent lanes are addressed in such two-dimensional periodic torus. Red, yellow, blue and green lines correspond to lanes ${\rm{i}}-2$, ${\rm{i}}-1$, i and ${\rm{i}}+1$. Arrows show allowed hopping, while cross displays prohibited one.

The sketch of the model is displayed in Fig. 1. Two-dimensional periodic boundaries and random update rules are applied, which consists of K equal-sized periodic subsystems. Each system size is L. As the periodic boundary, lane ${\rm{i}}+{\rm{K}}$ is equivalent to i. Additionally, ${\rm{K}} > 2$ is satisfied because each subsystem has two adjacent lanes. Here, a binary parameter ${{\rm{\tau }}}_{{\rm{i}},{\rm{j}}}({\rm{i}}=1\ldots {\rm{K}},{\rm{j}}=1\ldots {\rm{L}})$ is defined to illustrate the state of chosen site. In an infinitesimal time interval dt, if ${{\rm{\tau }}}_{{\rm{i}},{\rm{j}}}=1$ and ${{\rm{\tau }}}_{{\rm{i}}-1,{\rm{j}}}=0$, the chosen particle in lane i can move into corresponding site of ${\rm{i}}-1$ at ${{\rm{\omega }}}_{{\rm{i}}}^{{\rm{u}}}$. Similarly, if ${{\rm{\tau }}}_{{\rm{i}},{\rm{j}}}=1$ and ${{\rm{\tau }}}_{{\rm{i}}+1,{\rm{j}}}=0$, the chosen particle can move into ${\rm{i}}+1$ at ${{\rm{\omega }}}_{{\rm{i}}}^{{\rm{d}}}$. Thus, as the effect of hard-core exclusions, lane-changing behavior won’t occur until the target site is empty. Besides, if ${{\rm{\tau }}}_{{\rm{i}},{\rm{j}}}=1$ and ${{\rm{\tau }}}_{{\rm{i}},{\rm{j}}+1}=0$, the chosen particle can hop forward at p_i_. Moreover, totally heterogeneous interactions are considered, which satisfy ${{\rm{\omega }}}_{{\rm{i}}}^{{\rm{u}}}\ne {{\rm{\omega }}}_{{\rm{i}}}^{{\rm{d}}}$. Thus, three rates are coupled with each subsystem, namely upward rate ${{\rm{\omega }}}_{{\rm{i}}}^{{\rm{u}}}$, downward rate ${{\rm{\omega }}}_{{\rm{i}}}^{{\rm{d}}}$ and forward rate p_i_.

As the update rule, boundary of each subsystem is periodic in forward direction. Thus, dynamics of lane i are spatially independent, which mean that sites of it are equivalent to each other. Moreover, straight density profile of each subsystem also reflects that dynamics of each subsystem are spatially independent. Thus, dynamics of each subsystem are mainly controlled by heterogeneous interactions among adjacent subsystems. In the global view, dynamics of the proposed driven diffusive system are mainly affected by heterogeneous interactions, since they are capable of quantitatively reflecting interactions among adjacent subsystems and will affect the system’s local density. Additionally, in the special case ${{\rm{\omega }}}_{{\rm{i}}}^{{\rm{u}}}={{\rm{\omega }}}_{{\rm{i}}}^{{\rm{d}}}$, such totally heterogeneous system deteriorates into the partly heterogeneous one^39^. Moreover, in another special case that all transition rates are equal to each other, such totally heterogeneous system evolves into the homogeneous one.

### Detailed balance analysis

We consider particle configurations and use ${\{{{\rm{\tau }}}_{{\rm{i}},{\rm{j}}}\}}_{{\rm{i}}}$ to depict particle configurations in subsystem i. Similarly, f_i_ is set as the possibility of presence of particles on lattices in i. Because of homogeneity of each subsystem, possibilities can be expressed with the same f_i_ for each site. Then, the system’s partition function can be expressed as:

1 $${{\rm{Z}}}_{{\rm{L}},{\rm{N}},{\rm{K}}}=\sum \,{\prod }_{{\rm{i}}=1}^{{\rm{K}}}\,{{\rm{\psi }}}_{{\rm{i}}}({{\rm{M}}}_{{\rm{i}}}){\rm{f}}({\{{{\rm{\tau }}}_{{\rm{i}},{\rm{j}}}\}}_{{\rm{i}}}),$$

where N denotes total particle number. Besides, ${{\rm{\psi }}}_{{\rm{i}}}({{\rm{M}}}_{{\rm{i}}}){\rm{f}}({\{{{\rm{\tau }}}_{{\rm{i}},{\rm{j}}}\}}_{{\rm{i}}})$ means the probability of occurrence of the situation where subsystem i contains M_i_ particles with ${\{{{\rm{\tau }}}_{{\rm{i}},{\rm{j}}}\}}_{{\rm{i}}}$. Thus, ${\prod }_{{\rm{i}}=1}^{{\rm{K}}}{{\rm{\psi }}}_{{\rm{i}}}({{\rm{M}}}_{{\rm{i}}}){\rm{f}}({\{{{\rm{\tau }}}_{{\rm{i}},{\rm{j}}}\}}_{{\rm{i}}})$ means the probability of occurrence of the situation where those N particles distribute as $\{{{\rm{\tau }}}_{{\rm{i}},{\rm{j}}}\}$.

As for subsystem i, each particle has the same property. Therefore, subsystem i can be treated as a homogeneous system. Weight of different ${\{{{\rm{\tau }}}_{{\rm{i}},{\rm{j}}}\}}_{{\rm{i}}}$ should be equal with each other as for specific M_i_. Besides, it’s also equal to weight of occurrence of the situation where M_i_ sites are occupied in subsystem i. Thus, following restraint is satisfied:

2 $${{\rm{\psi }}}_{{\rm{i}}}({{\rm{M}}}_{{\rm{i}}}){\rm{f}}({\{{{\rm{\tau }}}_{{\rm{i}},{\rm{j}}}\}}_{{\rm{i}}})={{\rm{f}}}_{{\rm{i}}}^{{{\rm{M}}}_{{\rm{i}}}}.$$

In this circumstance, the weight can be divided into ${{\rm{C}}}_{{\rm{L}}}^{{{\rm{M}}}_{{\rm{i}}}}$ species. Then, Eq. (2) can be rewritten as:

3 $${{\rm{Z}}}_{{\rm{L}},{\rm{N}},{\rm{K}}}={\sum }_{{{\rm{M}}}_{1}=0}^{L}\,\ldots \,{\sum }_{{{\rm{M}}}_{{\rm{K}}}=0}^{L}\,{\prod }_{{\rm{i}}=1}^{{\rm{K}}}\,{{\rm{f}}}_{{\rm{i}}}^{{{\rm{M}}}_{{\rm{i}}}}(\begin{array}{c}L\\ {{\rm{M}}}_{{\rm{i}}}\end{array})\times \delta ({\sum }_{i=1}^{K}\,{{\rm{M}}}_{{\rm{i}}}-N).$$

As for a given configuration $\{{{\rm{\tau }}}_{{\rm{i}},{\rm{j}}}\}$, the probability P$\{{{\rm{\tau }}}_{{\rm{i}},{\rm{j}}}\}$ satisfies:

4 $${\rm{P}}\{{{\rm{\tau }}}_{{\rm{i}},{\rm{j}}}\}={Z}_{{\rm{L}},{\rm{N}},{\rm{K}}}^{-1}\,{\prod }_{{\rm{i}}=1}^{{\rm{K}}}\,{\rm{f}}({\{{{\rm{\tau }}}_{{\rm{i}},{\rm{j}}}\}}_{{\rm{i}}})={Z}_{{\rm{L}},{\rm{N}},{\rm{K}}}^{-1}\,{\prod }_{{\rm{i}}=1}^{{\rm{K}}}\,{{\rm{f}}}_{{\rm{i}}}^{{{\rm{M}}}_{{\rm{i}}}},$$

where ${\sum }_{{\rm{i}}}{\sum }_{{\rm{j}}}{\rm{P}}\{{{\rm{\tau }}}_{{\rm{i}},{\rm{j}}}\}=1$. Because the sum $\sum {\rm{P}}\{{{\rm{\tau }}}_{{\rm{i}},{\rm{j}}}\}$ of all probabilities of occurrence of all configurations is complete.

Besides, master equation is introduced to illustrate configuration transitions:

5 $$\frac{\partial P(C)}{\partial t}={\sum }_{C^{\prime} \ne C}\,\{P(C^{\prime} ){\rm{W}}(C^{\prime} \to C)-P(C){\rm{W}}(C\to C^{\prime} )\}=0,$$

where *C* and *C*′ denote the configuration before and after transition respectively. *P*(*C*) denotes the probability of occurrence of such configuration *C*. ${\rm{W}}(C\to C^{\prime} )$ indicates the probability of transition from C to C′. Since particles can perform both hopping in each lane or changing into the other lane, four states ${{\rm{C}}}_{1}^{^{\prime} }$, ${{\rm{C}}}_{1}^{^{\prime\prime} }$, ${{\rm{C}}}_{2}^{^{\prime} }$ and ${{\rm{C}}}_{2}^{^{\prime\prime} }$ are defined. In detail, both ${{\rm{C}}}_{1}^{^{\prime} }$ and ${{\rm{C}}}_{1}^{^{\prime\prime} }$ indicate the configuration caused by lane-switching. While, both ${{\rm{C}}}_{2}^{^{\prime} }$ and ${{\rm{C}}}_{2}^{^{\prime\prime} }$ reflect the one caused by hopping in the bulk. Moreover, both ${{\rm{C}}}_{1}^{^{\prime} }$ and ${{\rm{C}}}_{2}^{^{\prime} }$ mean the configuration after transition, which is generated from the original state C. While, ${{\rm{C}}}_{1}^{^{\prime\prime} }$ and ${{\rm{C}}}_{2}^{^{\prime\prime} }$ mean the configuration before transition, which will lead to the state C. Therefore, Eq. (5) can be rewritten as:

6 $$\begin{array}{c}{\sum }_{{{\rm{C}}}_{1}^{^{\prime} }}\,P(C)W(C\to {{\rm{C}}}_{1}^{^{\prime} })-{\sum }_{{{\rm{C}}}_{1}^{^{\prime\prime} }}\,P({{\rm{C}}}_{1}^{^{\prime\prime} })W({{\rm{C}}}_{1}^{^{\prime\prime} }\to {\rm{C}})+{\sum }_{{{\rm{C}}}_{2}^{^{\prime} }}\,P(C)W(C\to {{\rm{C}}}_{2}^{^{\prime} })\\ \,-{\sum }_{{{\rm{C}}}_{2}^{^{\prime\prime} }}\,P({{\rm{C}}}_{2}^{^{\prime\prime} })W({{\rm{C}}}_{2}^{^{\prime\prime} }\to {\rm{C}})=0.\end{array}$$

In fact, as for given ${{\rm{C}}}_{2}^{^{\prime} }$ and ${{\rm{C}}}_{2}^{^{\prime\prime} }$, $W(C\to {{\rm{C}}}_{2}^{^{\prime} })=W({{\rm{C}}}_{2}^{^{\prime\prime} }\to {\rm{C}})$ is satisfied, because both $W(C\to {{\rm{C}}}_{2}^{^{\prime} })$ and $W({{\rm{C}}}_{2}^{^{\prime\prime} }\to {\rm{C}})$ are equal to the hopping rate in the bulk. Besides, as the symmetry of topology of the proposed model, the number of ${{\rm{C}}}_{2}^{^{\prime} }$ should be equal to the one of ${{\rm{C}}}_{2}^{^{\prime\prime} }$. Moreover, as for a given ${{\rm{C}}}_{2}^{^{\prime\prime} }$, $P(C)=P({{\rm{C}}}_{2}^{^{\prime\prime} })$ is satisfied. Because the difference between C and ${{\rm{C}}}_{2}^{^{\prime\prime} }$ is just the specific location of particles while the number of particles remains unchanged. Thus, following equation can be derived:

7 $$\{\begin{array}{rcl}{\sum }_{{{\rm{C}}}_{2}^{^{\prime} }}P(C)W(C\to {{\rm{C}}}_{2}^{^{\prime} })-{\sum }_{{{\rm{C}}}_{2}^{^{\prime\prime} }}P({{\rm{C}}}_{2}^{^{\prime\prime} })W({{\rm{C}}}_{2}^{^{\prime\prime} }\to {\rm{C}}) & = & 0\\ {\sum }_{{{\rm{C}}}_{1}^{^{\prime} }}P(C)W(C\to {{\rm{C}}}_{1}^{^{\prime} })-{\sum }_{{{\rm{C}}}_{1}^{^{\prime\prime} }}P({{\rm{C}}}_{1}^{^{\prime\prime} })W({{\rm{C}}}_{1}^{^{\prime\prime} }\to {\rm{C}}) & = & 0\end{array}.$$

Furthermore, based on update rule and symmetry of the system, any state generated from C can also lead to C by performing lane-changing behavior, which means that each ${{\rm{C}}}_{1}^{^{\prime} }$ satisfies ${{\rm{C}}}_{1}^{^{\prime\prime} }$. Similarly, each ${{\rm{C}}}_{1}^{^{\prime\prime} }$ also satisfies ${{\rm{C}}}_{1}^{^{\prime} }$. Thus, both ${{\rm{C}}}_{1}^{^{\prime} }$ and ${{\rm{C}}}_{1}^{^{\prime\prime} }$ have the same physical meaning, which means that they are equivalent. Thus, the second expression in Eq. (7) can be rewritten as:

8 $${\sum }_{{{\rm{C}}}_{1}^{^{\prime} }}\,P(C)W(C\to {{\rm{C}}}_{1}^{^{\prime} })-{\sum }_{{{\rm{C}}}_{1}^{^{\prime} }}P({{\rm{C}}}_{1}^{^{\prime} })W({{\rm{C}}}_{1}^{^{\prime} }\to {\rm{C}})=0.$$

**Figure 2:**
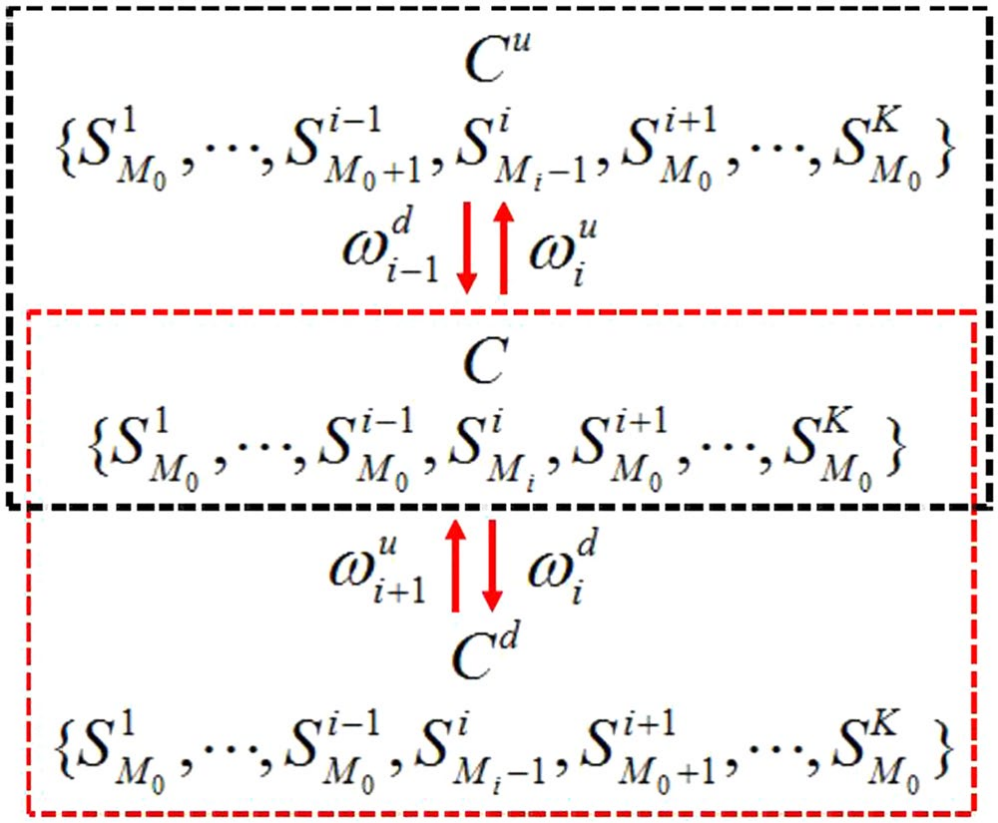
Transition between ${\rm{C}}(\{{{\rm{S}}}_{{{\rm{M}}}_{0}}^{1},\cdots ,{{\rm{S}}}_{{{\rm{M}}}_{0}}^{{\rm{i}}-1},{{\rm{S}}}_{{{\rm{M}}}_{{\rm{i}}}}^{{\rm{i}}},{{\rm{S}}}_{{{\rm{M}}}_{0}}^{{\rm{i}}+1},\cdots ,{{\rm{S}}}_{{{\rm{M}}}_{0}}^{{\rm{K}}}\})$ and other states. Transitions among C, ${{\rm{C}}}^{{\rm{u}}}(\{{{\rm{S}}}_{{{\rm{M}}}_{0}}^{1},\cdots ,{{\rm{S}}}_{{{\rm{M}}}_{0}+1}^{{\rm{i}}-1},{{\rm{S}}}_{{{\rm{M}}}_{{\rm{i}}}-1}^{{\rm{i}}},{{\rm{S}}}_{{{\rm{M}}}_{0}}^{{\rm{i}}+1},\cdots ,{{\rm{S}}}_{{{\rm{M}}}_{0}}^{{\rm{K}}}\})$ and ${{\rm{C}}}^{{\rm{d}}}(\{{{\rm{S}}}_{{{\rm{M}}}_{0}}^{1},\cdots ,{{\rm{S}}}_{{{\rm{M}}}_{0}}^{{\rm{i}}-1},{{\rm{S}}}_{{{\rm{M}}}_{{\rm{i}}}-1}^{{\rm{i}}},{{\rm{S}}}_{{{\rm{M}}}_{0}+1}^{{\rm{i}}+1},\cdots ,{{\rm{S}}}_{{{\rm{M}}}_{0}}^{{\rm{K}}}\})$ are addressed, which are shown in black and red boxes. C^*u*^ corresponds to ${\{{{\rm{\tau }}}_{{\rm{i}},{\rm{j}}}\}}^{u}$. C^*d*^ corresponds to ${\{{{\rm{\tau }}}_{{\rm{i}},{\rm{j}}}\}}^{d}$. ${{\rm{S}}}_{{\rm{M}}}^{{\rm{i}}}$ denotes lane *i* contains *M* particles with configuration ${\{{{\rm{\tau }}}_{{\rm{i}},{\rm{j}}}\}}_{i}$.

Here, we consider a specific situation. *C* is set to satisfy ${\{{{\rm{\tau }}}_{1,{\rm{j}}}\}}_{1}={\{{{\rm{\tau }}}_{2,{\rm{j}}}\}}_{2}=\cdots ={\{{{\rm{\tau }}}_{{\rm{i}}-1,{\rm{j}}}\}}_{{\rm{i}}-1}={\{{{\rm{\tau }}}_{{\rm{i}}+1,{\rm{j}}}\}}_{{\rm{i}}+1}=\cdots ={\{{{\rm{\tau }}}_{{\rm{K}},{\rm{j}}}\}}_{{\rm{K}}}$. Besides, the particle number in i is set to be more than that of any rest lanes, which means that ${{\rm{\tau }}}_{{\rm{i}},{\rm{j}}}=1$ and ${{\rm{\tau }}}_{{\rm{s}},{\rm{j}}}=1$ are satisfied for arbitrary ${\rm{j}}\in \{1\ldots {\rm{L}}\}$ and ${\rm{s}}\in \{1\ldots {\rm{i}}-1,{\rm{i}}+1\ldots {\rm{K}}\}$. While, there’s at least one value of h (${\rm{h}}\in \{1\ldots {\rm{L}}\}$) which satisfies ${{\rm{\tau }}}_{{\rm{i}},{\rm{h}}}=1$ and ${{\rm{\tau }}}_{{\rm{s}},{\rm{h}}}=0$. Thus, particles in the lane $1\ldots {\rm{i}}-1,{\rm{i}}+1\ldots {\rm{K}}$ cannot perform the lane-changing behavior. While, corresponding particles in i can hop into adjacent lanes. Here, Fig. 2 is applied to explain corresponding states before and after such transition. In this way, as for *C*, corresponding probability ${\rm{P}}(\{{{\rm{\tau }}}_{{\rm{i}},{\rm{j}}}\})$ becomes:

9 $${\rm{P}}(\{{{\rm{\tau }}}_{{\rm{i}},{\rm{j}}}\})={Z}_{{\rm{L}},{\rm{N}},{\rm{K}}}^{-1}{\rm{f}}({\{{{\rm{\tau }}}_{{\rm{i}},{\rm{j}}}\}}_{{\rm{i}}})={Z}_{{\rm{L}},{\rm{N}},{\rm{K}}}^{-1}{\,f}_{i}^{{M}_{i}}\,{\prod }_{j\ne i}\,{f}_{j}^{{M}_{0}},$$

where *M*_0_ means the number of rest of particles distributed in lane $1\ldots {\rm{i}}-1,{\rm{i}}+1\ldots {\rm{K}}$. Similarly, as for ${{\rm{C}}}_{1}^{^{\prime} }$, corresponding probability can be expressed as:

10 $$\{\begin{array}{rcl}{\rm{P}}({\{{{\rm{\tau }}}_{{\rm{i}},{\rm{j}}}\}}^{u}) & = & {Z}_{{\rm{L}},{\rm{N}},{\rm{K}}}^{-1}{\,f}_{i}^{{M}_{i}-1}{f}_{i-1}^{{M}_{0}+1}\,{\prod }_{j\ne i,i-1}\,{f}_{j}^{{M}_{0}}\\ {\rm{P}}({\{{{\rm{\tau }}}_{{\rm{i}},{\rm{j}}}\}}^{d}) & = & {Z}_{{\rm{L}},{\rm{N}},{\rm{K}}}^{-1}{\,f}_{i}^{{M}_{i}-1}{f}_{i+1}^{{M}_{0}+1}\,{\prod }_{j\ne i,i+1}\,{f}_{j}^{{M}_{0}}\end{array}.$$

**Figure 3:**
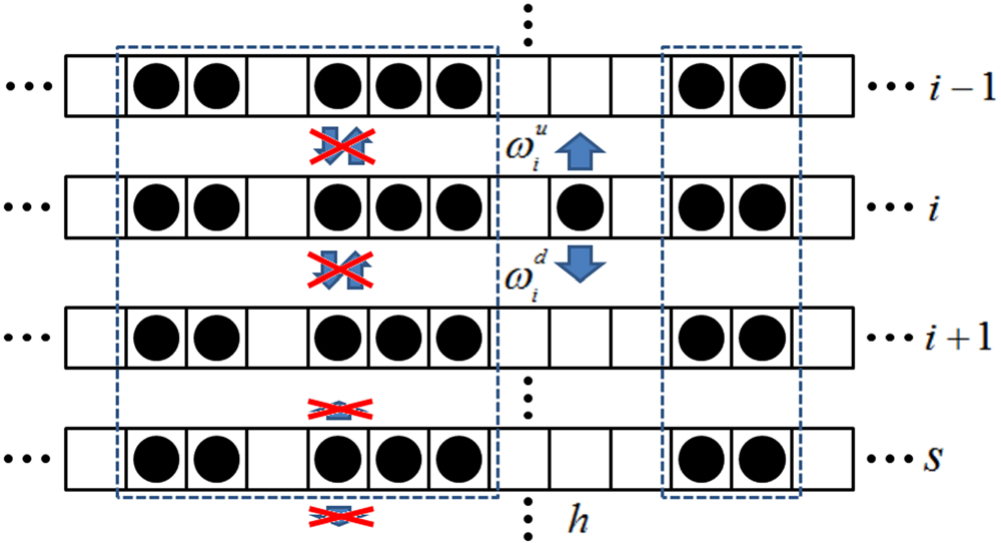
Lane-switching behavior among adjacent subsystems. h denotes the concerned site. Dashed box indicates the same configuration of transition. Arrows show allowed hopping, while cross displays prohibited one.

Additionally, Eq. (10) correspond to the situation where the particle in lane i moves into ${\rm{i}}-1$ and ${\rm{i}}+1$. Actually, both ${\{{{\rm{\tau }}}_{{\rm{i}},{\rm{j}}}\}}^{u}$ and ${\{{{\rm{\tau }}}_{{\rm{i}},{\rm{j}}}\}}^{d}$ are generated from C. Moreover, ${\{{{\rm{\tau }}}_{{\rm{i}},{\rm{j}}}\}}^{u}$ and ${\{{{\rm{\tau }}}_{{\rm{i}},{\rm{j}}}\}}^{d}$ indicate the configuration after transition that a particle in i updating into ${\rm{i}}-1$ and ${\rm{i}}+1$, respectively. Furthermore, the intuitive description of detailed balance is displayed in Fig. 3. By performing detailed balance analysis (see Methods), we can obtain:

11 $${f}_{i+1}{\omega }_{i+1}^{u}+{f}_{i-1}{\omega }_{i-1}^{d}-{f}_{i}{\omega }_{i}^{u}-{f}_{i}{\omega }_{i}^{d}=0.$$

Therefore, by solving linear equations composed by Eq. (11) (see Methods), the density weight can be derived:

12 $${f}_{i}=\frac{1}{2K{\omega }_{i}^{d}}(1+{\sum }_{j=1}^{K-1}{\prod }_{m=1}^{j}\,\frac{{\omega }_{i+m}^{u}}{{\omega }_{i+m}^{d}})+\frac{1}{2K{\omega }_{i}^{u}}(1+{\sum }_{j=1}^{K-1}{\prod }_{m=1}^{j}\,\frac{{\omega }_{i+K-m}^{d}}{{\omega }_{i+K-m}^{u}}).$$

Then, ${f}_{i}=1/\omega$ can be obtained from Eq. (12) when ${\omega }_{i}^{u}={\omega }_{i}^{d}=\omega$, which means that we have developed and generalized pre-existing results (namely, ref.^39^).

### Analytical and simulation results of characteristic parameters

By using complex analysis like intermediate value theorem etc. (see Methods), analytical results of characteristic order parameters are obtained. In details, global density $\rho$ can be got:

13 $$\rho =\frac{{\rm{N}}}{{\rm{L}}}={\sum }_{{\rm{i}}=1}^{{\rm{K}}}\,\tfrac{{\rm{z}}\,[\tfrac{1}{2K{\omega }_{i}^{d}}(1+{\sum }_{j=1}^{K-1}{\prod }_{m=1}^{j}\,\tfrac{{\omega }_{i+m}^{u}}{{\omega }_{i+m}^{d}})+\tfrac{1}{2K{\omega }_{i}^{u}}(1+{\sum }_{j=1}^{K-1}{\prod }_{m=1}^{j}\,\tfrac{{\omega }_{i+K-m}^{d}}{{\omega }_{i+K-m}^{u}})]}{1+{\rm{z}}\,[\tfrac{1}{2K{\omega }_{i}^{d}}(1+{\sum }_{j=1}^{K-1}{\prod }_{m=1}^{j}\,\tfrac{{\omega }_{i+m}^{u}}{{\omega }_{i+m}^{d}})+\tfrac{1}{2K{\omega }_{i}^{u}}(1+{\sum }^{K-1}{\prod }_{m=1}^{j}\,\tfrac{{\omega }_{i+K-m}^{d}}{{\omega }_{i+K-m}^{u}})]},$$

where z means root of Eq. (13). As for a specific system, $\rho$ can be determined since L, K and N are preset. Thus, solutions of z can be obtained from Eq. (13). Then, local density ${\rho }_{i}$ can be got:

14 $${{\rm{\rho }}}_{{\rm{i}}}=\tfrac{{\rm{z}}\,[\tfrac{1}{2K{\omega }_{i}^{d}}(1+{\sum }_{j=1}^{K-1}\,{\prod }_{m=1}^{j}\,\tfrac{{\omega }_{i+m}^{u}}{{\omega }_{i+m}^{d}})+\tfrac{1}{2K{\omega }_{i}^{u}}(1+{\sum }_{j=1}^{K-1}\,{\prod }_{m=1}^{j}\,\tfrac{{\omega }_{i+K-m}^{d}}{{\omega }_{i+K-m}^{u}})]}{1+{\rm{z}}\,[\tfrac{1}{2K{\omega }_{i}^{d}}(1+{\sum }_{j=1}^{K-1}\,{\prod }_{m=1}^{j}\,\tfrac{{\omega }_{i+m}^{u}}{{\omega }_{i+m}^{d}})+\tfrac{1}{2K{\omega }_{i}^{u}}(1+{\sum }_{j=1}^{K-1}\,{\prod }_{m=1}^{j}\,\tfrac{{\omega }_{i+K-m}^{d}}{{\omega }_{i+K-m}^{u}})]}{\rm{.}}$$

In fact, ${\rm{\rho }}$ is heavily dependent on ${{\rm{\rho }}}_{{\rm{i}}}$. Similarly, local current J_i_ can be given:

15 $${{\rm{J}}}_{{\rm{i}}}={p}_{{\rm{i}}}\tfrac{{\rm{z}}\,[\tfrac{1}{2K{\omega }_{i}^{d}}(1+{\sum }_{j=1}^{K-1}\,{\prod }_{m=1}^{j}\,\tfrac{{\omega }_{i+m}^{u}}{{\omega }_{i+m}^{d}})+\tfrac{1}{2K{\omega }_{i}^{u}}(1+{\sum }_{j=1}^{K-1}\,{\prod }_{m=1}^{j}\,\tfrac{{\omega }_{i+K-m}^{d}}{{\omega }_{i+K-m}^{u}})]}{{\{1+{\rm{z}}[\tfrac{1}{2K{\omega }_{i}^{d}}(1+{\sum }_{j=1}^{K-1}{\prod }_{m=1}^{j}\tfrac{{\omega }_{i+m}^{u}}{{\omega }_{i+m}^{d}})+\tfrac{1}{2K{\omega }_{i}^{u}}(1+{\sum }_{j=1}^{K-1}{\prod }_{m=1}^{j}\tfrac{{\omega }_{i+K-m}^{d}}{{\omega }_{i+K-m}^{u}})]\}}^{2}}.$$

Additionally, expectation $\langle {{\rm{n}}}_{{\rm{i}}}\rangle$ of particle number can be obtained:

16 $$\langle {{\rm{n}}}_{{\rm{i}}}\rangle =\tfrac{{\rm{zL}}[\tfrac{1}{2K{\omega }_{i}^{d}}(1+{\sum }_{j=1}^{K-1}\,{\prod }_{m=1}^{j}\,\tfrac{{\omega }_{i+m}^{u}}{{\omega }_{i+m}^{d}})+\tfrac{1}{2K{\omega }_{i}^{u}}(1+{\sum }_{j=1}^{K-1}\,{\prod }_{m=1}^{j}\,\tfrac{{\omega }_{i+K-m}^{d}}{{\omega }_{i+K-m}^{u}})]}{1+{\rm{z}}\,[\tfrac{1}{2K{\omega }_{i}^{d}}(1+{\sum }_{j=1}^{K-1}\,{\prod }_{m=1}^{j}\,\tfrac{{\omega }_{i+m}^{u}}{{\omega }_{i+m}^{d}})+\tfrac{1}{2K{\omega }_{i}^{u}}(1+{\sum }_{j=1}^{K-1}\,{\prod }_{m=1}^{j}\,\tfrac{{\omega }_{i+K-m}^{d}}{{\omega }_{i+K-m}^{u}})]}.$$

Furthermore, variance ${\rm{D}}\langle {{\rm{n}}}_{{\rm{i}}}\rangle$ of particle number can be obtained:

17 $${\rm{D}}\langle {{\rm{n}}}_{{\rm{i}}}\rangle =\tfrac{{\rm{zL}}[\tfrac{1}{2K{\omega }_{i}^{d}}(1+{\sum }_{j=1}^{K-1}\,{\prod }_{m=1}^{j}\,\tfrac{{\omega }_{i+m}^{u}}{{\omega }_{i+m}^{d}})+\tfrac{1}{2K{\omega }_{i}^{u}}(1+{\sum }_{j=1}^{K-1}\,{\prod }_{m=1}^{j}\,\tfrac{{\omega }_{i+K-m}^{d}}{{\omega }_{i+K-m}^{u}})]}{{\{1+{\rm{z}}[\tfrac{1}{2K{\omega }_{i}^{d}}(1+{\sum }_{j=1}^{K-1}{\prod }_{m=1}^{j}\tfrac{{\omega }_{i+m}^{u}}{{\omega }_{i+m}^{d}})+\tfrac{1}{2K{\omega }_{i}^{u}}(1+{\sum }_{j=1}^{K-1}{\prod }_{m=1}^{j}\tfrac{{\omega }_{i+K-m}^{d}}{{\omega }_{i+K-m}^{u}})]\}}^{2}}.$$

As for each subsystem, the probability of having particles or no particles at any two positions is the same. According to the law of large numbers, $\frac{{{\rm{n}}}_{{\rm{i}}}}{{\rm{L}}}$ converges to ${{\rm{\rho }}}_{{\rm{i}}}$ in probability when L is large enough, which can be expressed as:

18 $${\rm{P}}(|\frac{{{\rm{n}}}_{{\rm{i}}}}{{\rm{L}}}-{{\rm{\rho }}}_{{\rm{i}}}| > \delta ) < \varepsilon .$$

Here, Eq. (18) is satisfied for arbitrary positive number δ and ${\rm{\varepsilon }}$. Finally, effects of fully heterogeneous interactions on totally current and global transport are emphasized. Based on Eq. (15), the maximum value J_max_ of the total current J_total_ can be derived:

19 $${{\rm{J}}}_{{\rm{\max }}}={\sum }_{{\rm{i}}=1}^{{\rm{K}}}\,(0.25\,({{\rm{p}}}_{{\rm{i}}}\,{\sum }_{{\rm{i}}=1}^{{\rm{K}}}\,\tfrac{1}{{{\rm{p}}}_{{\rm{i}}}}-0.5\,{\rm{K}}+\tfrac{{\rm{N}}}{{\rm{L}}})\,({{\rm{p}}}_{{\rm{i}}}\,{\sum }_{{\rm{i}}=1}^{{\rm{K}}}\,\tfrac{1}{{{\rm{p}}}_{{\rm{i}}}}+0.5\,{\rm{K}}-\tfrac{{\rm{N}}}{{\rm{L}}})/({{\rm{p}}}_{{\rm{i}}}{({\sum }_{{\rm{i}}=1}^{{\rm{K}}}\tfrac{1}{{{\rm{p}}}_{{\rm{i}}}})}^{2}))$$

under extreme condition:

20 $$\tfrac{{\rm{z}}\,[\tfrac{1}{2K{\omega }_{i}^{d}}(1+{\sum }_{j=1}^{K-1}\,{\prod }_{m=1}^{j}\,\tfrac{{\omega }_{i+m}^{u}}{{\omega }_{i+m}^{d}})+\tfrac{1}{2K{\omega }_{i}^{u}}(1+{\sum }_{j=1}^{K-1}\,{\prod }_{m=1}^{j}\,\tfrac{{\omega }_{i+K-m}^{d}}{{\omega }_{i+K-m}^{u}})]}{1+{\rm{z}}\,[\tfrac{1}{2K{\omega }_{i}^{d}}(1+{\sum }_{j=1}^{K-1}\,{\prod }_{m=1}^{j}\,\tfrac{{\omega }_{i+m}^{u}}{{\omega }_{i+m}^{d}})+\tfrac{1}{2K{\omega }_{i}^{u}}(1+{\sum }_{j=1}^{K-1}\,{\prod }_{m=1}^{j}\,\tfrac{{\omega }_{i+K-m}^{d}}{{\omega }_{i+K-m}^{u}})]}=\tfrac{0.5({p}_{i}{\sum }_{i=1}^{K}\,\tfrac{1}{{p}_{i}}-0.5K+\tfrac{N}{L})}{{p}_{i}\,{\sum }_{i=1}^{K}\,\tfrac{1}{{p}_{i}}}.$$

**Figure 4:**
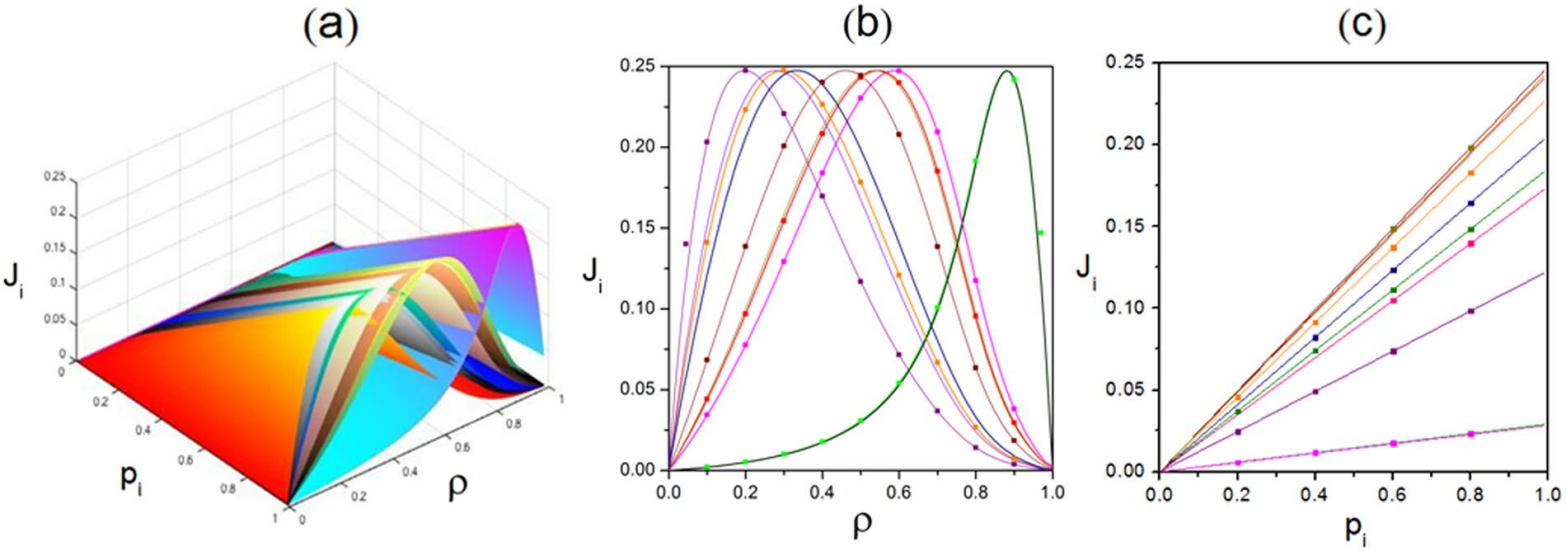
Relationship among the global density ${\rm{\rho }}$, forward rate p_i_ and local current J_i_. (**a**) Three-dimensional view. (**b**) Plot of J_i_ versus ${\rm{\rho }}$ under ${{\rm{p}}}_{{\rm{i}}}=1$. (**c**) Plot of J_i_ versus p_i_ under ${\rm{\rho }}=0.5$. Scatters are Monte-Carlo simulations. Lines are analytical results. Parameters are $K=10$, $\,{\omega }_{1}^{u}=0.005$, ${\omega }_{1}^{d}=0.384$, ${\omega }_{2}^{u}=0.156$, ${\omega }_{2}^{d}=0.437$, ${\omega }_{3}^{u}=0.305$, ${\omega }_{3}^{d}=0.482$, ${\omega }_{4}^{u}=0.457$, ${\omega }_{4}^{d}=0.034$, ${\omega }_{5}^{u}=0.103$, ${\omega }_{5}^{d}=0.083$, ${\omega }_{6}^{u}=0.254$, ${\omega }_{6}^{d}=0.136$, ${\omega }_{7}^{u}=0.404$, ${\omega }_{7}^{d}=0.187$, ${\omega }_{8}^{u}=0.056$, ${\omega }_{8}^{d}=0.233$, ${\omega }_{9}^{u}=0.208$, ${\omega }_{9}^{d}=0.285$, ${\omega }_{10}^{u}=0.359$ and ${\omega }_{10}^{d}=0.335$.

**Figure 5:**
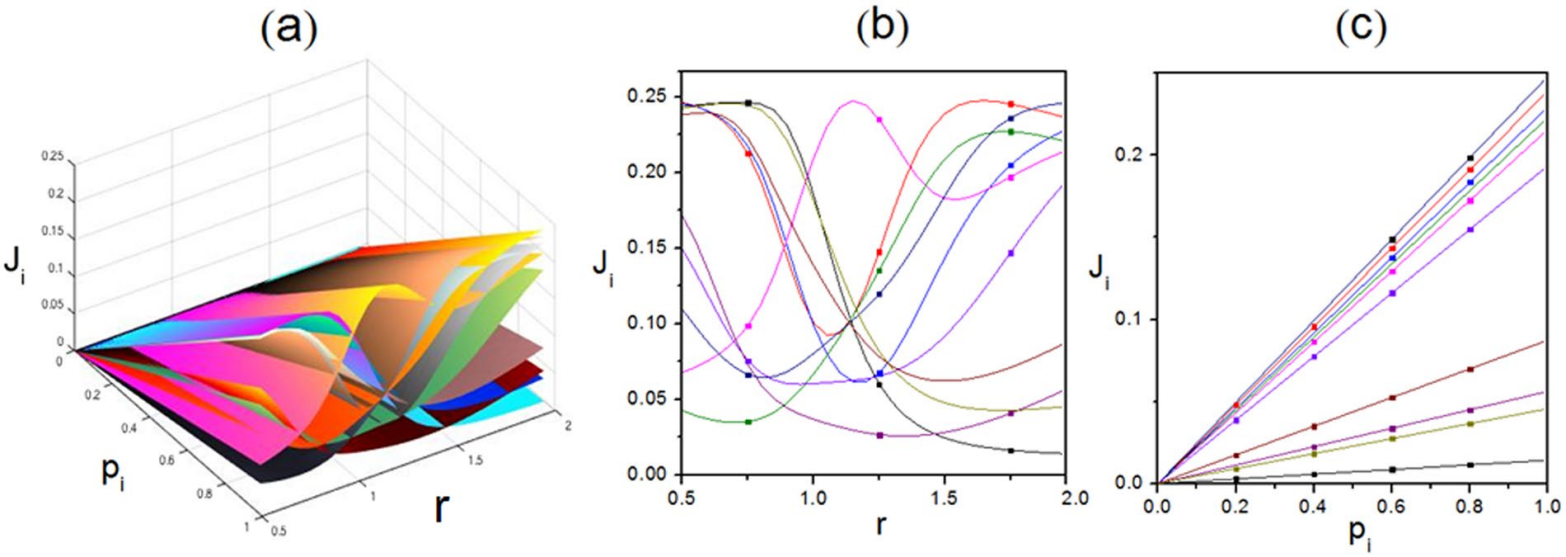
Relationship among the local current J_i_, scaling rate r and forward rate p_i_. (**a**) Three-dimensional view. (**b**) Plot of J_i_ versus r under ${{\rm{p}}}_{{\rm{i}}}=1$. (**c**) Plot of J_i_ versus p_i_ under ${\rm{r}}=1.95$. Scatters are Monte-Carlo simulations. Lines are analytical results. Parameters are $K=10\,$and ${\rm{\rho }}=0.5$. Primitive transition rates are $\,{\omega }_{1}^{u}=0.005$, ${\omega }_{1}^{d}=0.384$, ${\omega }_{2}^{u}=0.156$, ${\omega }_{2}^{d}=0.437$, ${\omega }_{3}^{u}=0.305$, ${\omega }_{3}^{d}=0.482$, ${\omega }_{4}^{u}=0.457$, ${\omega }_{4}^{d}=0.034$, ${\omega }_{5}^{u}=0.103$, ${\omega }_{5}^{d}=0.083$, ${\omega }_{6}^{u}=0.254$, ${\omega }_{6}^{d}=0.136$, ${\omega }_{7}^{u}=0.404$, ${\omega }_{7}^{d}=0.187$, ${\omega }_{8}^{u}=0.056$, $\,{\omega }_{8}^{d}=0.233$, ${\omega }_{9}^{u}=0.208$, ${\omega }_{9}^{d}=0.285$, ${\omega }_{10}^{u}=0.359$ and ${\omega }_{10}^{d}=0.335$. Upward and downward rates of each lane are $r$ and 1/*r* times of original values respectively.

**Figure 6:**
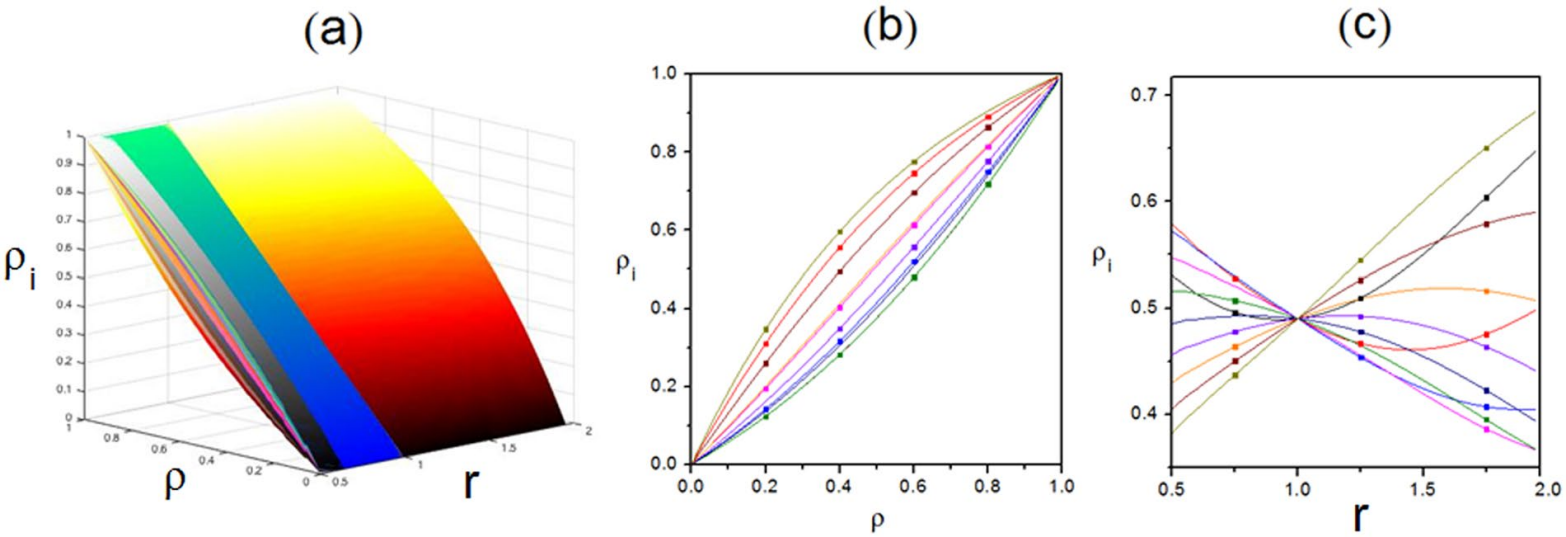
Relationship among the global density ${\rm{\rho }}$, local density ${{\rm{\rho }}}_{{\rm{i}}}$ and scaling rate **r** with fixed primitive transition rates. (**a**) Three-dimensional view. (**b**) Plot of ${{\rm{\rho }}}_{{\rm{i}}}$ versus ${\rm{\rho }}$ under ${\rm{r}}=1.95$. (**c**) Plot of ${{\rm{\rho }}}_{{\rm{i}}}$ versus r under ${\rm{\rho }}=0.5$. Scatters are Monte-Carlo simulations. Lines are analytical results. Parameters are $K=10,\,\,{\omega }_{i}^{u}=0.15{r}^{\frac{i-1}{10}}$ and $\,{\omega }_{i}^{d}=0.35{r}^{\frac{1-i}{10}}$.

**Figure 7:**
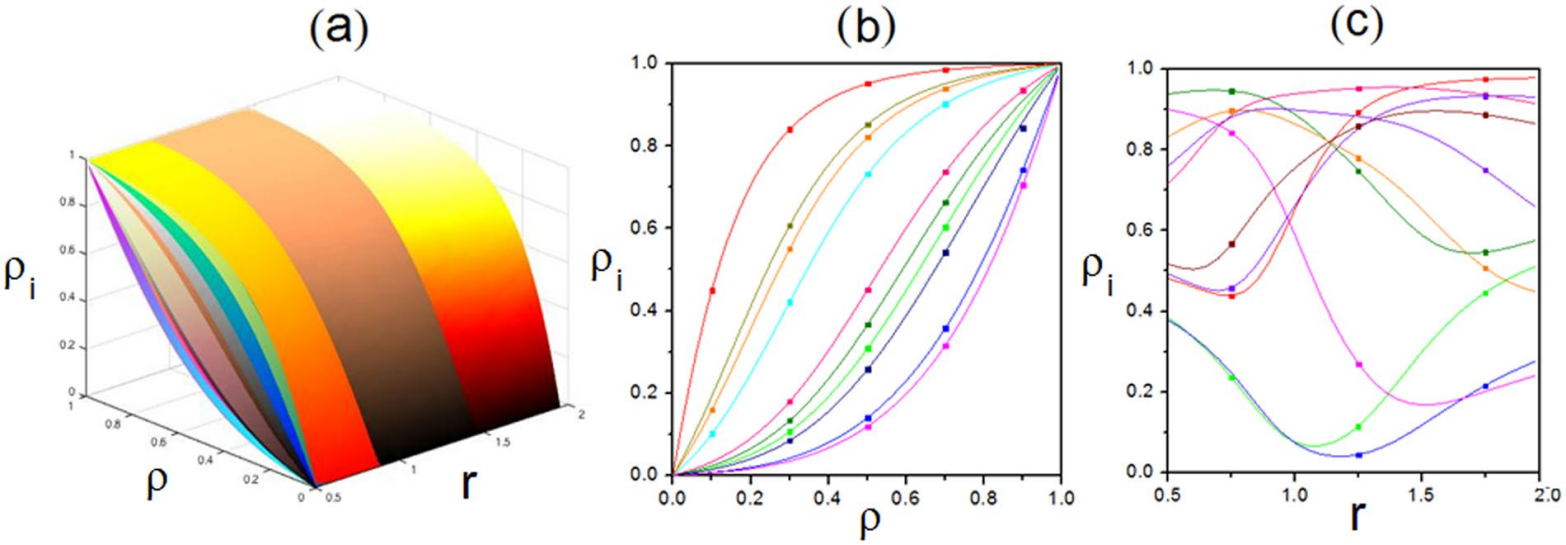
Relationship among the global density ${\rm{\rho }}$, local density ${{\rm{\rho }}}_{{\rm{i}}}$ and scaling rate r with random primitive transition rates. (**a**) Three-dimensional view. (**b**) Plot of ${{\rm{\rho }}}_{{\rm{i}}}$ versus ${\rm{\rho }}$ under ${\rm{r}}=1.95$. (**c**) Plot of ${{\rm{\rho }}}_{{\rm{i}}}$ versus ${\rm{r}}$ under ${\rm{\rho }}=0.5$. Scatters are Monte-Carlo simulations. Lines are analytical results. $K$ takes $10$. Primitive transition rates are $\,{\omega }_{1}^{u}=0.005$, ${\omega }_{1}^{d}=0.384$, ${\omega }_{2}^{u}=0.156$, ${\omega }_{2}^{d}=0.437$, ${\omega }_{3}^{u}=0.305$, ${\omega }_{3}^{d}=0.482$, ${\omega }_{4}^{u}=0.457$, ${\omega }_{4}^{d}=0.034$, ${\omega }_{5}^{u}=0.103$, ${\omega }_{5}^{d}=0.083$, ${\omega }_{6}^{u}=0.254$, ${\omega }_{6}^{d}=0.136$, ${\omega }_{7}^{u}=0.404$, ${\omega }_{7}^{d}=0.187$, ${\omega }_{8}^{u}=0.056$, ${\omega }_{8}^{d}=0.233$, ${\omega }_{9}^{u}=0.208$, ${\omega }_{9}^{d}=0.285$, ${\omega }_{10}^{u}=0.359$ and ${\omega }_{10}^{d}=0.335$. Upward and downward rates of each subsystem are $r\,$and ${r}^{-1}$ times of original values respectively.

**Figure 8:**
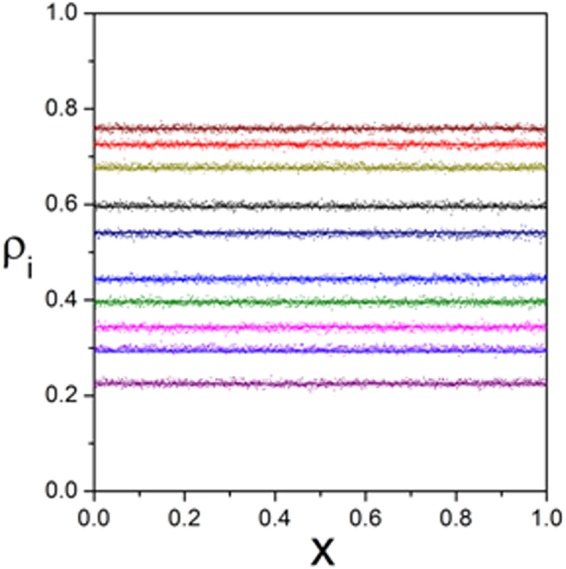
Density profiles ${{\rm{\rho }}}_{{\rm{i}}}({\rm{x}})$ of each subsystem. Lines depict analytical results. Scatters reflect Monte-Carlo simulations. Parameters are $K=10$, ${\omega }_{1}^{u}=0.273$, ${\omega }_{1}^{d}=0.431$, ${\omega }_{2}^{u}=0.123$, ${\omega }_{2}^{d}=0.172$, ${\omega }_{3}^{u}=0.039$, ${\omega }_{3}^{d}=0.47$, ${\omega }_{4}^{u}=0.448$, ${\omega }_{4}^{d}=0.155$, ${\omega }_{5}^{u}=0.23$, ${\omega }_{5}^{d}=0.432$, ${\omega }_{6}^{u}=0.065$, ${\omega }_{6}^{d}=0.171$, ${\omega }_{7}^{u}=0.179$, ${\omega }_{7}^{d}=0.297$, ${\omega }_{8}^{u}=0.379,\,{\omega }_{8}^{d}=0.43$, ${\omega }_{9}^{u}=0.098,\,{\omega }_{9}^{d}=0.051,\,\,{\omega }_{10}^{u}=0.279$ and ${\omega }_{10}^{d}=0.17$.

**Figure 9:**
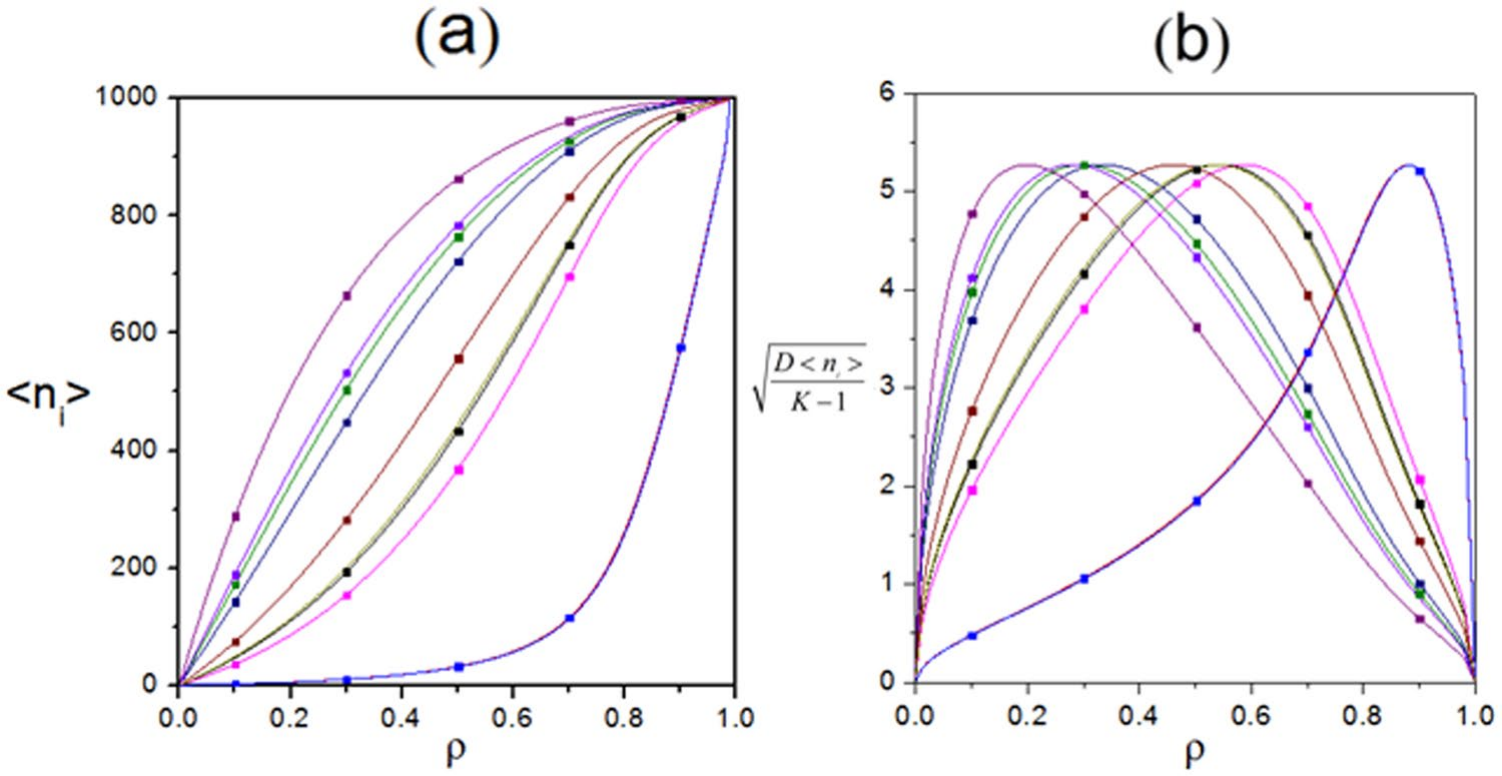
Plot of expectation and standard deviation of particle numbers in subsystem changing with global density. (**a**) Expectation $$\langle {{\rm{n}}}_{{\rm{i}}}\rangle $$. (**b**) Standard deviation $$\sqrt{{\rm{D}}\langle {{\rm{n}}}_{{\rm{i}}}\rangle /(K-1)}$$. Lines depict analytical results. Scatters reflect Monte-Carlo simulations. Parameters are $$K=10,\,{\omega }_{1}^{u}=0.005,\,{\omega }_{1}^{d}=0.384$$, $${\omega }_{2}^{u}=0.156,\,{\omega }_{2}^{d}=0.437,\,{\omega }_{3}^{u}=0.305$$, $${\omega }_{3}^{d}=0.482,\,{\omega }_{4}^{u}=0.457,\,{\omega }_{4}^{d}=0.034$$, $${\omega }_{5}^{u}=0.103,\,{\omega }_{5}^{d}=0.083,\,{\omega }_{6}^{u}=0.254$$, $${\omega }_{6}^{d}=0.136,\,{\omega }_{7}^{u}=0.404,$$$${\omega }_{7}^{d}=0.187$$, $${\omega }_{8}^{u}=0.056,\,{\omega }_{8}^{d}=0.233,\,{\omega }_{9}^{u}=0.208$$, $${\omega }_{9}^{d}=0.285,\,{\omega }_{10}^{u}=0.359$$ and $${\omega }_{10}^{d}=0.335$$.

**Figure 10:**
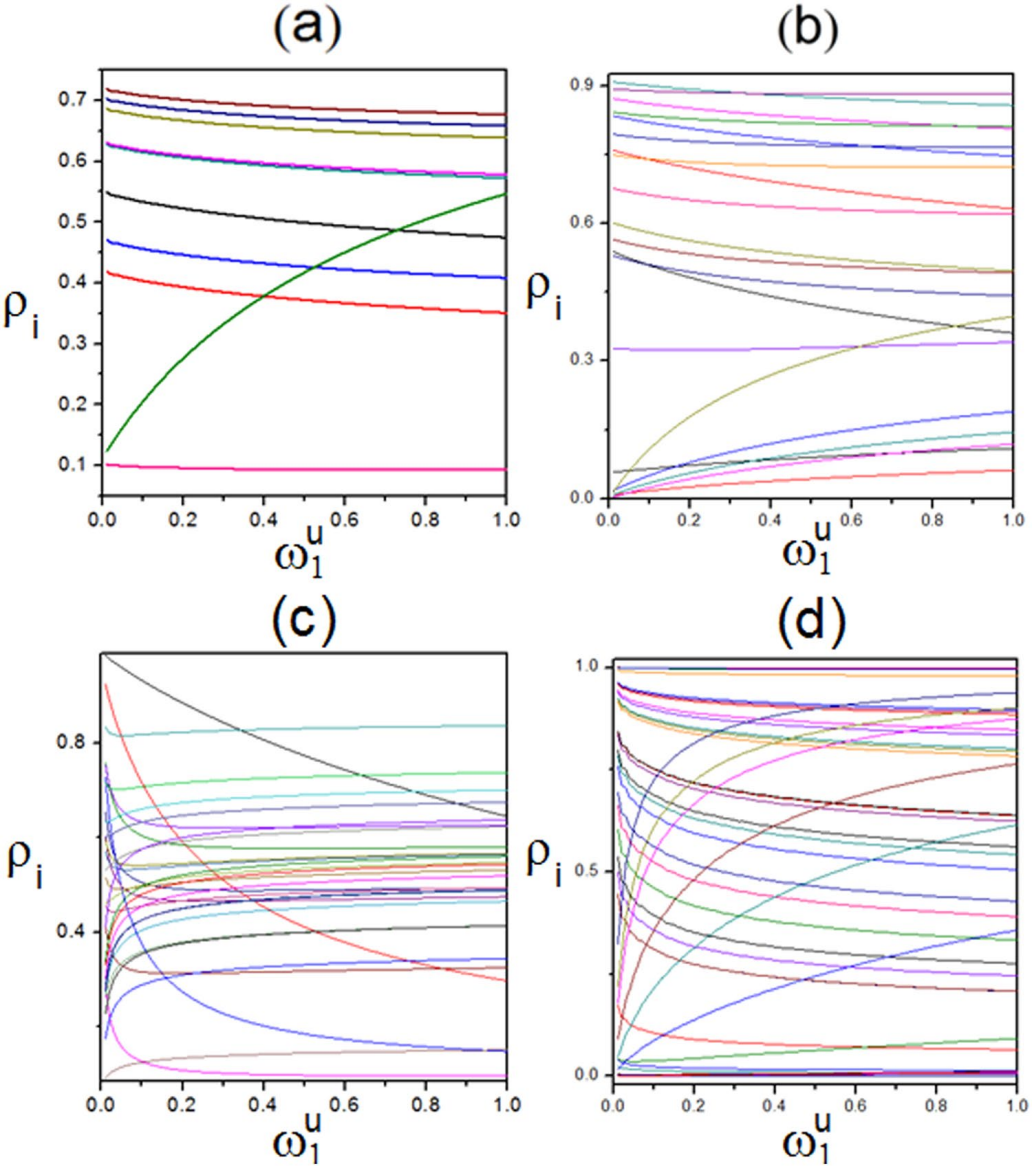
Relationship among channel numbers *K*, local density ${{\rm{\rho }}}_{{\rm{i}}}$ and transition rate ${\omega }_{1}^{u}$. (**a**) $K=10$. (**b**) $K=20$. (**c**) $K=30$. (**d**) $K=50$. Parameters are$\,{\rm{\rho }}=0.5$ and randomly generated transition rates ranging from 0 to 1.

**Figure 11:**
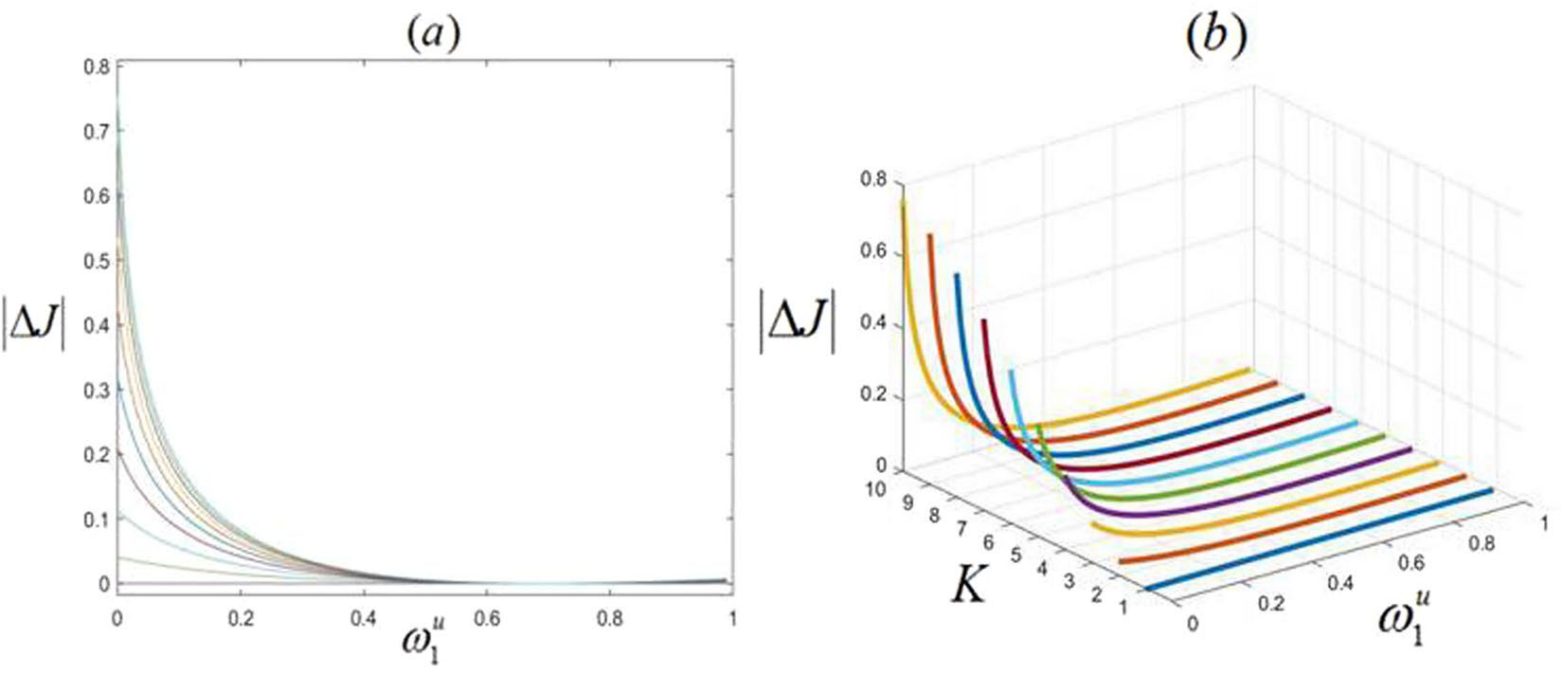
Relationship among the absolute value $|{\rm{\Delta }}J|$ of increment of total current, channel number ${\rm{K}}$ and transition rate ${{\rm{\omega }}}_{1}^{{\rm{u}}}$. (**a**) $|{\rm{\Delta }}J|$ versus ${{\rm{\omega }}}_{1}^{{\rm{u}}}$. (**b**) Three-dimensional view. ${\rm{\rho }}$ takes 0.5. Upward and downward rates of each subsystem are 0.15 and 0.35 respectively.

**Figure 12:**
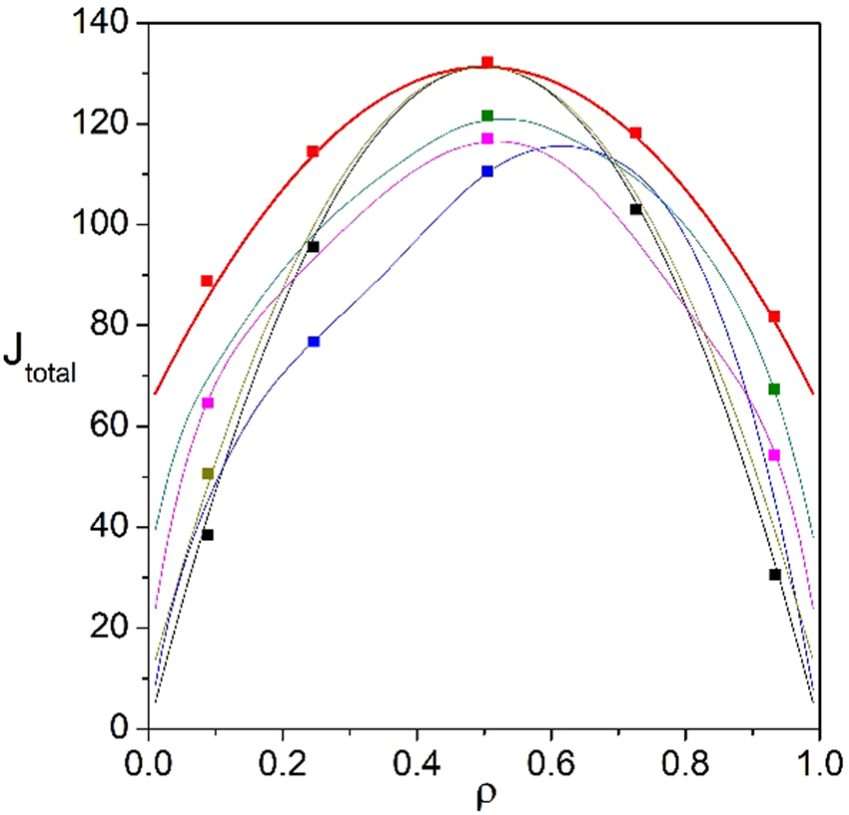
Relationship between global density ${\rm{\rho }}$ and total current J_total_ in the comparison of three complete kinds of situations. Red line shows the optimal J_total_ in our work. Dark yellow line corresponds to homogeneous interactions. Magenta line shows a typical case of partly heterogeneous interactions (namely, ref.^39^). Black and blue lines depict other typical cases of partly heterogeneous interactions (namely, equal inner and outside interactions between adjacent TASEPs, respectively). Dark cyan represents the universal case of partly heterogeneous interactions. Scatters are Monte-Carlo simulations. Parameters are ${\rm{K}}=100$, ${\rm{L}}=200$ and randomly generated p_i_.

**Figure 13:**
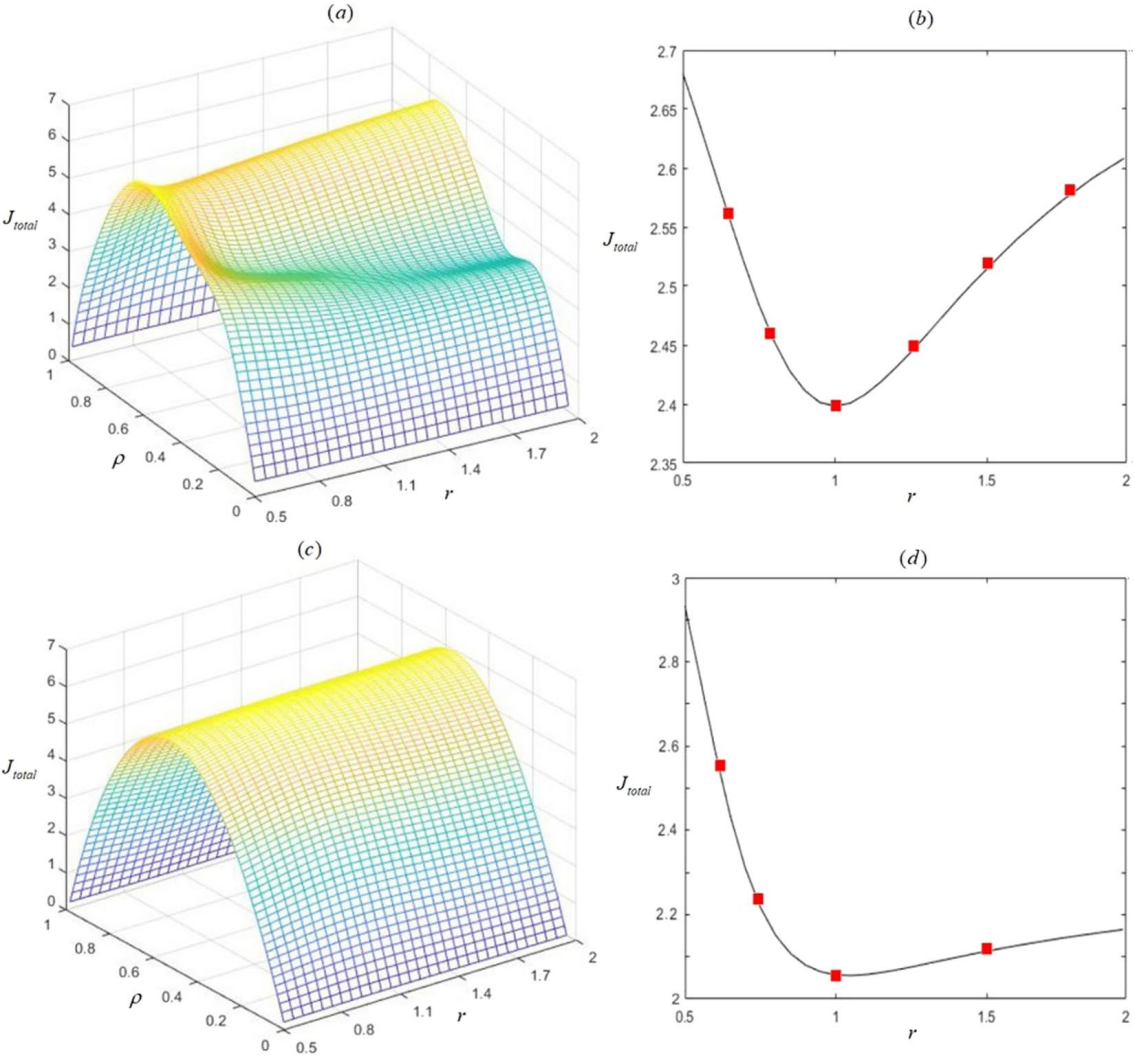
Relationship among total current J_total_, global density ${\rm{\rho }}$ and scaling rate r in the comparison of three complete kinds of situations. (**a**) Intuitively three-dimensional view of comparison of our work and the universal case of partly heterogeneous. (**b**) Plot of J_total_ versus r under ${\rm{\rho }}=0.9$ in the comparison shown in (**a**). (**c**) Intuitively three-dimensional view of comparison of our work and homogeneous. (**d**) Plot of J_total_ versus r under ${\rm{\rho }}=0.9$ in the comparison shown in (**c**). $\,{\omega }_{i}^{u}$ and $\,{\omega }_{i}^{d}$ of the pre-selected subsystem *i* for (**a**,**b**) are ${{\rm{r}}}^{\frac{{\rm{i}}}{1000}}$ and ${{\rm{r}}}^{-\frac{{\rm{i}}}{1000}}$ times of randomly generated primitive transition rates. While, corresponding transition rates of other unselected subsystems are randomly generated. Besides, ${\omega }_{i}^{u}$ and ${\omega }_{i}^{d}$ for (**c**,**d**) are $0.3{{\rm{r}}}^{\frac{{\rm{i}}}{1000}}$ and $0.3{{\rm{r}}}^{-\frac{{\rm{i}}}{1000}}$ respectively. Scatters depict Monte-Carlo simulations. Lines depict analytical analyses. Parameters are ${\rm{K}}=100$, ${\rm{L}}=200$ and randomly generated p_i_.

Additionally, both analytical and Monte-Carlo simulation results of characteristic order parameters are shown in Figs 4, 5, 6, 7, 8, 9, 10, 11, 12 and 13. In Monte-Carlo simulations for Figs 4, 5, 6, 7, 8, 9, 10 and 11, L takes 1000. The number of total time steps T is set as 10^10^ to obtain steady state. Besides, the final 90% of time steps are retained to ensure occurrence of steady state. In details, Fig. 4 shows relationship among ${\rm{\rho }}$, *p*_*i*_ and J_i_. It reveals that *J*_*i*_ varies for a given ${\rm{\rho }}$ because of heterogeneous interactions among subsystems. However, as the capacity of maximum current of TASEP, *J*_*i*_ is not monotonously changing with ${\rm{\rho }}$. Moreover, the value of ${\rm{\rho }}$ corresponding to peak value of *J*_*i*_ is also varied for each subsystem as such heterogeneous interactions. The maximum current in each subsystem satisfies 0.25*p*_*i*_, which can also be revealed from the constraint ${J}_{i}={p}_{i}\,{\rho }_{i}(1-{\rho }_{i})$ by combining Eqs (14) and (15). Additionally, the current in each lane increases linearly with forward rate, which can be reflected from Fig. 4(c) and the above constraint. In details, heterogeneous interactions are reflected by asymmetric rates between adjacent subsystems, which can lead to various site occupation rates. Then, different cluster states of particles occur. Thus, varied particle stochastic dynamics of each specific lane are presented, including various densities, diverse flows etc.

In order to highlight the effect of transition rates on local current, the scaling rate *r* is introduced. Here, *r* denotes a factor that acts on preset transition rates and depicts the extent of heterogeneity of the global system. Firstly, primitive transition rates are set. Here, primitive transition rates denote the target that the scaling rate *r* acts on, which are preset and randomly generated. Three complete kinds of such driven diffusive system can be constructed, namely the case of totally heterogeneous interactions, partly heterogeneous ones and homogeneous one. Then, *r* acts on the primitive transition rate, which makes upward and downward transition rates become *r* and 1/*r* times of original values respectively. Figure 5 shows the relationship among J_i_, *r* and *p*_*i*_. It can be found that different channels exhibit various properties. Local current reaches the extremum with different scaling rates and contains disparate number of peaks. Since particles in the system have two degrees of freedom, the effect of forward motion also needs to be emphasized. Compared with Fig. 4, the relationship between J_i_ and *p*_*i*_ in Fig. 5 is similar to that reflected in Fig. 4 in terms of quantitative and qualitative evolution rules. This is because that configuration of particles in each subsystem can be determined for preset transition rates. Then, the bulk current is heavily dependent on unidirectional self-driven motions of particles. Thus, in this circumstance, the local current is proportional to forward rate.

Besides, Figs 6 and 7 display relationship among ${\rm{\rho }}$, ${{\rm{\rho }}}_{{\rm{i}}}$ and *r*. In Fig. 6, *r* affects each channel differently. Primitive transition rates are set as identical constants. ${{\rm{\rho }}}_{{\rm{i}}}$ is found to evolve in different ways as various effects of *r*. Particularly, ${{\rm{\rho }}}_{{\rm{i}}}$ is the same when ${\rm{r}}=1$, since r has no effect on transition rates in this circumstance. Moreover, as the influence of r, ${{\rm{\rho }}}_{{\rm{i}}}$ will be different with increasing ${\rm{\rho }}$. Additionally, as conserved particle number, ${{\rm{\rho }}}_{{\rm{i}}}$ also increases monotonously with ${\rm{\rho }}$. Especially, ${{\rm{\rho }}}_{{\rm{i}}}$ becomes 0 when ${\rm{\rho }}=0$, as there’re no particles. Similarly, as the full capacity, ${{\rm{\rho }}}_{{\rm{i}}}$ reaches 1 when ${\rm{\rho }}=1$. While, the case of randomly generated primitive transition rates is shown in Fig. 7 with the same effect of *r* on each subsystem. Compared with Fig. 6, similar evolution law of density is presented in the form of a spindle although different values of ${{\rm{\rho }}}_{{\rm{i}}}$ appear. It also reveals that all analytical results match well with Monte-Carlo simulations.

Then, corresponding density profiles ${{\rm{\rho }}}_{{\rm{i}}}({\rm{x}})$ are displayed in Fig. 8. It reveals that ${{\rm{\rho }}}_{{\rm{i}}}({\rm{x}})$ is uniform in space because ${{\rm{\rho }}}_{{\rm{i}}}({\rm{x}})$ is independent of the location x of sites as the periodic boundary condition and update rule of the proposed system, which means that any particle in i is equivalent to each other. In other words, the interaction among particles in i is homogeneous. Moreover, Fig. 9 shows $\langle {{\rm{n}}}_{{\rm{i}}}\rangle$ and $\sqrt{{\rm{D}}\langle {{\rm{n}}}_{{\rm{i}}}\rangle /(K-1)}$ changing with ${\rm{\rho }}$. Evolution rules of $\langle {{\rm{n}}}_{{\rm{i}}}\rangle$ and ${{\rm{\rho }}}_{{\rm{i}}}$ along with ${\rm{\rho }}$ are similar, since $\langle {{\rm{n}}}_{{\rm{i}}}\rangle ={\rm{L}}\,{{\rm{\rho }}}_{{\rm{i}}}$. However, that of $\sqrt{{\rm{D}}\langle {{\rm{n}}}_{{\rm{i}}}\rangle /(K-1)}$ is different due to various ${\omega }_{i}^{u}$ and ${\omega }_{i}^{d}$. In addition, the standard deviation first increases and then decreases with ${\rm{\rho }}$. Particularly, $\sqrt{{\rm{D}}\langle {{\rm{n}}}_{{\rm{i}}}\rangle /(K-1)}$ becomes 0 when ${\rm{\rho }}=0$ and becomes 1 when ${\rm{\rho }}=1$. Furthermore, peaks of $\sqrt{{\rm{D}}\langle {{\rm{n}}}_{{\rm{i}}}\rangle /(K-1)}$ are disparate as different ${\omega }_{i}^{u}$ and ${\omega }_{i}^{d}$.

Moreover, we aim at analyzing the influence of disturbance on the system coupled with changing transition rates. Thus, for simplicity, the upward transition rate ${\omega }_{1}^{u}$ of lane 1 is set to range from 0 to 1 here, while other transition rates except for ${\omega }_{1}^{u}$ remain unchanged. Corresponding transition rates are randomly generated to confirm validity of universality of calculations. More complex topologies (namely, $K=10,20,30,50$) rather than just previous four TASEPs^39^ are calculated. Relationship among *K*, ${{\rm{\rho }}}_{{\rm{i}}}$ and ${\omega }_{1}^{u}$ is displayed in Fig. 10. As for Fig. 10(a), it describes ${\rm{K}}=10$, where randomly generated transition rates except for ${{\rm{\omega }}}_{1}^{{\rm{u}}}$ range from 0 to 1. Ten colorful lines show evolvements of ${\rm{\rho }}$ with increasing ${{\rm{\omega }}}_{1}^{{\rm{u}}}$. Especially, green line displays lane 10, which reveals that ${{\rm{\rho }}}_{10}$ increases with ${{\rm{\omega }}}_{1}^{{\rm{u}}}$ and the gradient of change of it gradually decreases, since increasing ${{\rm{\omega }}}_{1}^{{\rm{u}}}$ directly leads to the increase of particle number in lane 10. Besides, black line shows lane 1, which shows that ${{\rm{\rho }}}_{1}$ decreases with ${{\rm{\omega }}}_{1}^{{\rm{u}}}$, since increasing ${{\rm{\omega }}}_{1}^{{\rm{u}}}$ directly leads to the decrease of particle number in lane 1. Similarly, Fig. 10(b) shows ${\rm{K}}=20$. Increasing ${{\rm{\omega }}}_{1}^{{\rm{u}}}$ directly leads to decreasing ${{\rm{\rho }}}_{1}$ and increasing ${{\rm{\rho }}}_{20}$. Besides, densities of other remained lanes also become varied. Moreover, Fig. 10(c) depicts ${\rm{K}}=30$. Similarly, increasing ${{\rm{\omega }}}_{1}^{{\rm{u}}}$ directly leads to decreasing ${{\rm{\rho }}}_{1}$ and increasing ${{\rm{\rho }}}_{30}$, which also causes changes of other subsystems. Furthermore, Fig. 10(d) illustrates ${\rm{K}}=50$. Similarly, increasing ${{\rm{\omega }}}_{1}^{{\rm{u}}}$ directly causes decreasing ${{\rm{\rho }}}_{1}$ and increasing ${{\rm{\rho }}}_{30}$, which also leads to changes of other channels.

Thus, based on Fig. 10, it reveals that no matter how system’s topology changes, the disturbance will spread and affect local densities of other channels, when transition rate of one channel changes. The first and K-th channels are the most affected since ${{\rm{\omega }}}_{1}^{{\rm{u}}}$ changes. Increasing ${{\rm{\omega }}}_{1}^{{\rm{u}}}$ leads to continuously decreasing ${{\rm{\rho }}}_{1}$ and increasing ${{\rm{\rho }}}_{{\rm{K}}}$. Additionally, except for lanes 1 and K, the disturbance will have greater impacts on lanes 2 and $({\rm{K}}-1)$ because of stronger interactions among them. Here, it should be addressed that as changing ${\omega }_{1}^{u}$ is set to illustrate the influence of disturbance, increasing ${\omega }_{1}^{u}$ will directly lead to decreasing ${{\rm{\rho }}}_{1}$ and increasing ${{\rm{\rho }}}_{{\rm{K}}}$. Thus, lanes 1 and K should be emphasized. Finally, Fig. 11 displays the relationship among the absolute value $|{\rm{\Delta }}J|$ of increment of total current, K and ${\omega }_{1}^{u}$. It reflects that $|{\rm{\Delta }}J|$ monotonically decreases for any channel number with increasing ${\omega }_{1}^{u}$. When ${\omega }_{1}^{u}$ degrades the system from heterogeneous one to homogeneous one, ${\rm{\Delta }}$J becomes 0. Moreover, total current increases monotonously with increasing channel number.

Additionally, besides in technical perspective, the improvement of our work compared with pre-existing results is intuitively presented through Figs 12 and 13. In details, beyond pre-existing results, the improvement of our work can be intuitively found in Fig. 12, through studying the relationship between ${\rm{\rho }}$ and J_total_ in three complete kinds of such driven diffusive system, namely totally heterogeneous interactions (namely, our work), partly heterogeneous ones and homogeneous ones. Under any conserved global density, the optimal total current obtained from our work is generally greater than that of homogeneous one or partly heterogeneous ones including pre-existing results^39,40^ and the universal case of partly heterogeneous. Besides, the value of optimal total current obtained from our model is equal to that of homogeneous when ${\rm{\rho }}=0.5$, since the optimal current in each channel reaches to the maximum. Here, the universal case of partly heterogeneous interactions in Fig. 12 is built by equal transition rates for some randomly chosen channels (namely, equal transition rates ${{\rm{\chi }}}_{i}$ for pre-selected channel *i* and ${{\rm{\chi }}}_{i}\ne {{\rm{\chi }}}_{j}$ for ∀*i* ≠ *j*) and randomly generated rates for remaining ones. Thus, improving pre-existing results, our work presents a method of optimizing overall transport of such driven-diffusive systems in the global perspective under conserved global density, since the optimal total current obtained from our work introducing and considering totally heterogeneous interactions is generally greater than that of homogeneous one or partly heterogeneous ones. Here, 100 TASEPs are considered in calculations to obtain enough large and universal driven-diffusive system to confirm the validity of the improvement. Moreover, matching well with analytical results, Monte-Carlo simulations are also performed to make sure the validity of the improvement of our work comparing with pre-existing results.

Moreover, in order to intuitively describe the improvement of our work compared with pre-existing results, the effect of introducing and considering heterogeneous interactions on overall transport is further studied by calculating the relationship among *J*_*total*_, $\rho$ and r shown in Fig. 13. Comparisons of our work with the universal case of partly heterogeneous are presented in Fig. 13(a,b), since totally heterogeneous case will evolve into the universal case of partly heterogeneous when ${\rm{r}}=1$. While, comparisons of our work with homogeneous case are displayed in Fig. 13(c,d), where totally heterogeneous case can evolve into homogeneous one when ${\rm{r}}=1$. Based on Fig. 13(a,c), it can be found that *J*_*total*_ increases at first and then decreases later with increasing $\rho$. However, based on Fig. 13(b,d), it reveals that the optimal current of our work is much higher than that under partially heterogeneous interactions and homogeneous one. Thus, our work also reveals that introducing and considering totally heterogeneous interactions can increase the optimal total current in overall transport of self-driven particles in such driven diffusive system modelled by multiple TASEPs, which can also reflect that totally heterogeneous interactions can improve the overall transport of such interacting multi-body particle systems.

Furthermore, based on Figs 4, 5, 6, 7, 8, 9, 10, 11, 12 and 13, it should be pointed out that heterogeneous multilane TASEPs proposed here can also be equivalent to driven diffusive system coupled with Langmuir dynamics of detachment rate 1/f_i_ and attachment rate z^12^. This is due to following reasons. Firstly, actually, these concerned multiple TASEPs can be treated as being in contact with the thermal bath that washes out any density-density correlation whereas in the case under scrutiny the global density is conserved. Then, in the view of statistical mechanics, proposed system can be treated as a grand canonical ensemble. Because each channel of our model can exchange particles with adjacent subsystems with conserved global density, which can fit well with the essence of grand canonical ensemble, namely, each system can exchange energy and particles with other systems with conserved overall potential. Hereafter, ref.^12^ reported that at high kinetic rates (namely, high values of attachment rate ${{\rm{\omega }}}_{{\rm{A}}}$ and detachment rate ${{\rm{\omega }}}_{{\rm{D}}}$) the bulk density in TASEP was structureless and equal to the Langmuir equilibrium density $\frac{{\rm{K}}}{{\rm{K}}+1}$, where the ratio K satisfied ${\rm{K}}=\frac{{{\rm{\omega }}}_{{\rm{A}}}}{{{\rm{\omega }}}_{{\rm{D}}}}$. That’s to say, when high values of the attachment and detachment rates are set^12^, bulk dynamics will dominate in the competition with boundary dynamics, which means that the model can degenerate into the periodic boundary condition. Thus, based on Langmuir equilibrium density $\frac{{\rm{K}}}{{\rm{K}}+1}$, Eqs (12) and (14), ${{\rm{\rho }}}_{{\rm{i}}}$ can be derived as ${{\rm{\rho }}}_{{\rm{i}}}=\frac{{{\rm{zf}}}_{{\rm{i}}}}{1+{{\rm{zf}}}_{{\rm{i}}}}=\frac{{\rm{K}}}{1+{\rm{K}}}$. Therefore, our model can be mapped into the one of ref.^12^ with ${\rm{K}}={{\rm{zf}}}_{{\rm{i}}}$, where equivalent kinetic rates satisfy ${{\rm{\omega }}}_{{\rm{A}}}={\rm{z}}$ and ${{\rm{\omega }}}_{{\rm{D}}}=\frac{1}{{f}_{i}}$.

## Discussion

To summarize, we propose a driven diffusive system composed of K TASEPs. Different from previous work, interactions among adjacent subsystems is asymmetric heterogeneous. Generally, the whole system is coupled with periodic boundaries in a two-dimensional periodic torus. Self-driven particles can switch into adjacent channels or unidirectionally move. System’s stochastic dynamics are mainly controlled by transition rates rather than hopping rate due to spatially homogeneous local densities. Based on detailed balance principle, we check master equation. Then, restraint about weight factor f_i_ is obtained ${f}_{i+1}{\omega }_{i+1}^{u}+{f}_{i-1}{\omega }_{i-1}^{d}-{f}_{i}{\omega }_{i}^{u}-{f}_{i}{\omega }_{i}^{d}=0$. Afterwards, by solving linear equations, f_i_ is got ${f}_{i}=\frac{1}{2K{\omega }_{i}^{d}}(1+{\sum }_{j=1}^{K-1}{\prod }_{m=1}^{j}\frac{{\omega }_{i+m}^{u}}{{\omega }_{i+m}^{d}})$ + $\frac{1}{2K{\omega }_{i}^{u}}(1+{\sum }_{j=1}^{K-1}{\prod }_{m=1}^{j}\frac{{\omega }_{i+K-m}^{d}}{{\omega }_{i+K-m}^{u}})$. Thus, the expression evolves into ${f}_{i}=1/\omega$ when ${\omega }_{i}^{u}={\omega }_{i}^{d}=\omega$, which proves that our model can spontaneously evolve into the system coupled with symmetric transition rates. That’s to say, we have generalized previous research work^39^. Moreover, by applying complex analysis, analytical results of characteristic order parameters are obtained, including ${\rho }_{i}$, *J*_*i*_, $\rho$, $\langle {{\rm{n}}}_{{\rm{i}}}\rangle$, ${\rm{D}}\langle {{\rm{n}}}_{{\rm{i}}}\rangle$ and probability of configuration $\frac{{{\rm{n}}}_{{\rm{i}}}}{{\rm{L}}}$.

Besides, universal characteristics are revealed by both analytical solutions and Monte-Carlo simulations which match well with each other. Local density is found to monotonously increase with global density. Due to totally heterogeneous interactions, specific local densities are different from each other. Based on homogeneity on spatial site locations, local density profiles are spatially homogeneous, which are reflected by horizontal lines. Moreover, due to capacity of the maximum flow in TASEP, the relationship between local current and global density doesn’t monotonously change, which appears different from that of densities. While, local current is proportional to it at given transition rates. Additionally, by calculating more complex topologies (namely, *K* = 10, 20, 30, 50) of such driven diffusive system rather than just previous four TASEPs^39^, it reveals that no matter how system’s topology changes, the disturbance of changing transition rate of one subsystem will spread and affect local densities of other subsystems. As asymmetric heterogeneous interactions among adjacent subsystems, changing interaction rate of any subsystem will directly lead to variations of densities of its adjacent channels, which also means a larger change in particle configurations in adjacent ones. Because the changing of spatially dependent local density is equivalent to changing of dynamics of particle configurations, since local density is the essentially statistical average value of occupation probability of sites in each subsystem. Moreover, as each lattice position with or without particles are independent events, expectation is equal to system size multiplied by local density. Similarly, variance is equal to system size multiplied by ${{\rm{\rho }}}_{{\rm{i}}}(1-{{\rm{\rho }}}_{{\rm{i}}})$. Thus, both expectation and variance depend on global density. With increasing global density, expectation monotonously increases, while variance first increases and then decreases.

Finally, in order to detailly describe the improvement of our work, we firstly interpret the previous work in this area and then detailly compare results of our work with pre-existing physical systems. Improving pre-existing results, we propose a more universal and realistic interacting multi-body particle system which is more reliable to depict real transport phenomena. Thus, we focus on constructing such driven-diffusive system by employing multi-channel TASEPs with totally heterogeneous interactions. Besides, we obtain explicit analytical solutions and Monte-Carlo simulations of such proposed driven-diffusive system to avoid using previous approximation methods that often lead to results lack of universal laws. Moreover, different with previous work, we get universal laws of characteristic order parameters of such proposed driven diffusive system by considering complex topological structures. Furthermore, we also point out that introducing and considering totally heterogeneous interactions in such driven-diffusive system can increase the optimal total current in overall transport of the global driven diffusive system, which can also reflect that totally heterogeneous interactions can improve the overall transport of such interacting multi-body particle systems. Besides, based on our research work, it can also be revealed that introducing and considering totally and fully heterogeneous interactions is a way to optimize the total current in such driven diffusive system modelled by multiple TASEPs under the circumstance of conserved global density, which can be proved by calculating the relationship between global density and total current in three complete kinds of situations (namely, our work, partly heterogeneous interactions and homogeneous), relationships among J_total_, ρ and r in the intuitively three-dimensional view and the relationship between J_total_ and r with fixed value of ρ in intuitively two-dimensional view. Additionally, we also point out totally heterogeneous interaction rates can improve the total current in such multiple TASEP system and optimize the overall transport of such driven-diffusive system. Our research will be helpful to study other homeomorphism systems like coupling with memory reservoirs, which will be helpful for better understanding of stochastic particle dynamics in driven diffusive systems constituted by multiple TASEPs. Other performing work will be reported later.

## Methods

### Detailed balance equation analysis

For simplicity, we use the symbol *A* to denote ${\sum }_{{{\rm{C}}}_{1}^{^{\prime} }}\,P(C)W(C\to {{\rm{C}}}_{1}^{^{\prime} })-{\sum }_{{{\rm{C}}}_{1}^{^{\prime} }}\,P({{\rm{C}}}_{1}^{^{\prime} })W({{\rm{C}}}_{1}^{^{\prime} }\to {\rm{C}})$. Based on Eqs (9) and (10), detailed balance equation can be derived:

21 $$\begin{array}{rcl}A & = & ({M}_{i}-{M}_{0}){\rm{P}}(\{{{\rm{\tau }}}_{{\rm{i}},{\rm{j}}}\})({\omega }_{i}^{u}+{\omega }_{i}^{d})-\,({M}_{i}-{M}_{0}){\rm{P}}({\{{{\rm{\tau }}}_{{\rm{i}},{\rm{j}}}\}}^{d}){\omega }_{i+1}^{u}\\  &  & -({M}_{i}-{M}_{0}){\rm{P}}({\{{{\rm{\tau }}}_{{\rm{i}},{\rm{j}}}\}}^{u}){\omega }_{i-1}^{d}\\  & = & ({M}_{i}-{M}_{0}){Z}_{{\rm{L}},{\rm{N}},{\rm{K}}}^{-1}({f}_{i}^{{M}_{i}}\,{\prod }_{j\ne i}\,{f}_{j}^{{M}_{0}}({\omega }_{i}^{u}+{\omega }_{i}^{d})\\  &  & -\,{f}_{i}^{{M}_{i}-1}{f}_{i-1}^{{M}_{0}+1}{\prod }_{j\ne i,i-1}\,{f}_{j}^{{M}_{0}}{\omega }_{i-1}^{d}-{f}_{i}^{{M}_{i}-1}{f}_{i+1}^{{M}_{0}+1}{\prod }_{j\ne i,i+1}\,{f}_{j}^{{M}_{0}}{\omega }_{i+1}^{u})\\  & = & ({M}_{i}-{M}_{0}){Z}_{{\rm{L}},{\rm{N}},{\rm{K}}}^{-1}{\,f}_{i}^{{M}_{i}-1}\,{\prod }_{j\ne i}\,{f}_{j}^{{M}_{0}}(\,{f}_{i}{\omega }_{i}^{u}+{f}_{i}{\omega }_{i}^{d}-{f}_{i+1}{\omega }_{i+1}^{u}-{f}_{i-1}{\omega }_{i-1}^{d})\\  & = & 0\end{array}$$

Thus, ${f}_{i+1}{\omega }_{i+1}^{u}+{f}_{i-1}{\omega }_{i-1}^{d}-{f}_{i}{\omega }_{i}^{u}-{f}_{i}{\omega }_{i}^{d}=0$ is obtained, which is Eq. (11).

### Solving linear equations composed by Equation (11)

In fact, Eq. (11) is satisfied for arbitrary $i\in \{1,2,\ldots ,K\}$. Thus, *K* similar equations like Eq. (11) can be obtained. Besides, these *K* equations constitute the following linear equations:

22 $$WF=0$$

where

$$W=(\begin{array}{cccccccc}-({{\rm{\omega }}}_{1}^{{\rm{u}}}+{{\rm{\omega }}}_{1}^{{\rm{d}}}) & {{\rm{\omega }}}_{2}^{{\rm{u}}} & 0 & 0 & \cdots  & 0 & 0 & {{\rm{\omega }}}_{{\rm{K}}}^{{\rm{d}}}\\ {{\rm{\omega }}}_{1}^{{\rm{d}}} & -({{\rm{\omega }}}_{2}^{{\rm{u}}}+{{\rm{\omega }}}_{2}^{{\rm{d}}}) & {{\rm{\omega }}}_{3}^{{\rm{u}}} & 0 & 0 & \cdots  & 0 & 0\\ 0 & {{\rm{\omega }}}_{2}^{{\rm{d}}} & -({{\rm{\omega }}}_{3}^{{\rm{u}}}+{{\rm{\omega }}}_{3}^{{\rm{d}}}) & {{\rm{\omega }}}_{4}^{{\rm{u}}} & 0 & 0 & \cdots  & 0\\ \vdots  & \vdots  &  &  &  &  &  & \vdots \\ {{\rm{\omega }}}_{1}^{{\rm{u}}} & 0 & 0 & 0 & \cdots  & 0 & {{\rm{\omega }}}_{{\rm{K}}-1}^{{\rm{d}}} & -({{\rm{\omega }}}_{{\rm{K}}}^{{\rm{u}}}+{{\rm{\omega }}}_{{\rm{K}}}^{{\rm{d}}})\end{array}){\rm{and}}$$

$F={({f}_{1}{f}_{2}\cdots {f}_{K})}^{T}$ are satisfied. Then, the rank of the matrix *W* needs to be investigated in order to solve Eq. (22). Additionally, the cofactor of *W* can be derived:

23 $$\,{W}_{11}={(-1)}^{{\rm{K}}-1}(\,{\sum }_{{\rm{j}}=0}^{{\rm{K}}-1}\,{\prod }_{{\rm{m}}=2+{\rm{j}}}^{{\rm{K}}}\,{{\rm{\omega }}}_{{\rm{m}}}^{{\rm{d}}}{\prod }_{{\rm{n}}=2}^{1+{\rm{j}}}\,{{\rm{\omega }}}_{{\rm{n}}}^{{\rm{u}}}).$$

Here, *W*_11_ denotes one of the cofactors, which corresponds to the element located in the first row and the first column of *W*. Thus, ${W}_{11}\ne 0$ is satisfied. Moreover, *W* is not full rank. Therefore, the rank of *W* is *K* − 1. That’s to say, the solution of Eq. (22) is one-dimensional.

Then, in order to solve Eq. (22), we suppose that following equation is satisfied for arbitrary $i\in \{1,2,\ldots ,K\}$:

24 $${B}_{i}=\frac{1}{2K{\omega }_{i}^{d}}(1+{\sum }_{j=1}^{K-1}{\prod }_{m=1}^{j}\,\frac{{\omega }_{i+m}^{u}}{{\omega }_{i+m}^{d}})+\frac{1}{2K{\omega }_{i}^{u}}(1+{\sum }_{j=1}^{K-1}{\prod }_{m=1}^{j}\,\frac{{\omega }_{i+K-m}^{d}}{{\omega }_{i+K-m}^{u}}).$$

Thus, $F={({B}_{1}{B}_{2}\cdots {B}_{K})}^{T}$ should be proved to be the solution of Eq. (22). As the symmetry of topology and updating rule, following constraints are satisfied:

25 $$\{\begin{array}{c}{\omega }_{m}^{d}={\omega }_{m+K}^{d}\\ {\omega }_{m}^{u}={\omega }_{m+K}^{u}\end{array}.$$

Afterwards, based on Eqs (24) and (25), the equation can be obtained:

26 $$\begin{array}{rcl}{B}_{i}{\omega }_{i}^{d}+{B}_{i}{\omega }_{i}^{u} & = & \tfrac{1}{2K}(1+{\sum }_{j=1}^{K-1}{\prod }_{m=1}^{j}\,\tfrac{{\omega }_{i+m}^{u}}{{\omega }_{i+m}^{d}})+\tfrac{1}{2K}{\sum }_{j=0}^{K-1}{\prod }_{m=0}^{j}\,\tfrac{{\omega }_{i+m}^{u}}{{\omega }_{i+m}^{u}}\\  &  & +\tfrac{1}{2K}(1+{\sum }_{j=1}^{K-1}{\prod }_{m=1}^{j}\,\tfrac{{\omega }_{i+K-m}^{d}}{{\omega }_{i+K-m}^{u}})+\tfrac{1}{2K}{\sum }_{j=0}^{K-1}{\prod }_{m=0}^{j}\,\tfrac{{\omega }_{i+K-m}^{d}}{{\omega }_{i+K-m}^{u}}\\  & = & \tfrac{1}{2K}+\tfrac{1}{2K}{\sum }_{j=1}^{K-1}{\prod }_{m=1}^{j}\,\tfrac{{\omega }_{i+m}^{u}}{{\omega }_{i+m}^{d}}\\  &  & +\tfrac{1}{2K}{\sum }_{j=0}^{K-2}{\prod }_{m=0}^{j}\,\tfrac{{\omega }_{i+m}^{u}}{{\omega }_{i+m}^{d}}+\tfrac{1}{2K}{\prod }_{m=0}^{K-1}\,\tfrac{{\omega }_{i+m}^{u}}{{\omega }_{i+m}^{d}}\\  &  & +\tfrac{1}{2K}+\tfrac{1}{2K}{\sum }_{j=1}^{K-1}{\prod }_{m=1}^{j}\,\tfrac{{\omega }_{i+K-m}^{d}}{{\omega }_{i+K-m}^{u}}\\  &  & +\tfrac{1}{2K}{\sum }_{j=0}^{K-2}{\prod }_{m=0}^{j}\,\tfrac{{\omega }_{i+K-m}^{d}}{{\omega }_{i+K-m}^{u}}+\tfrac{1}{2K}{\prod }_{m=0}^{K-1}\,\tfrac{{\omega }_{i+K-m}^{d}}{{\omega }_{i+K-m}^{u}}\\  & = & \tfrac{1}{2K}(1+{\sum }_{j=0}^{K-2}{\prod }_{m=0}^{j}\,\tfrac{{\omega }_{i+m}^{u}}{{\omega }_{i+m}^{d}})+\tfrac{1}{2K}{\sum }_{j=1}^{K}{\prod }_{m=1}^{j}\,\tfrac{{\omega }_{i+m}^{u}}{{\omega }_{i+m}^{d}}\\  &  & +\tfrac{1}{2K}(1+{\sum }_{j=0}^{K-2}{\prod }_{m=0}^{j}\,\tfrac{{\omega }_{i+K-m}^{d}}{{\omega }_{i+K-m}^{u}})+\tfrac{1}{2K}{\sum }_{j=1}^{K}{\prod }_{m=1}^{j}\,\tfrac{{\omega }_{i+K-m}^{d}}{{\omega }_{i+K-m}^{u}}\\  & = & \tfrac{1}{2K}(1+{\sum }_{j=1}^{K-1}{\prod }_{m=1}^{j}\,\tfrac{{\omega }_{i-1+m}^{u}}{{\omega }_{i-1+m}^{d}})+\tfrac{1}{2K}{\sum }_{j=0}^{K-1}{\prod }_{m=0}^{j}\,\tfrac{{\omega }_{i+1+m}^{u}}{{\omega }_{i+1+m}^{d}}\\  &  & +\tfrac{1}{2K}(1+{\sum }_{j=1}^{K-1}{\prod }_{m=1}^{j}\,\tfrac{{\omega }_{i+K+1-m}^{d}}{{\omega }_{i+K+1-m}^{u}})+\tfrac{1}{2K}{\sum }_{j=0}^{K-1}{\prod }_{m=0}^{j}\,\tfrac{{\omega }_{i-1+K-m}^{d}}{{\omega }_{i-1+K-m}^{u}}\\  & = & [\tfrac{1}{2K{\omega }_{i-1}^{d}}(1+{\sum }_{j=1}^{K-1}{\prod }_{m=1}^{j}\,\tfrac{{\omega }_{i-1+m}^{u}}{{\omega }_{i-1+m}^{d}})\\  &  & +\,\tfrac{1}{2K{\omega }_{i-1}^{u}}(1+{\sum }_{j=1}^{K-1}{\prod }_{m=1}^{j}\,\tfrac{{\omega }_{i-1+K-m}^{d}}{{\omega }_{i-1+K-m}^{u}})]{\omega }_{i-1}^{d}\\  &  & +\,[\tfrac{1}{2K{\omega }_{i+1}^{d}}(1+{\sum }_{j=1}^{K-1}{\prod }_{m=1}^{j}\,\tfrac{{\omega }_{i+1+m}^{u}}{{\omega }_{i+1+m}^{d}})\\  &  & +\,\tfrac{1}{2K{\omega }_{i+1}^{u}}(1+{\sum }_{j=1}^{K-1}{\prod }_{m=1}^{j}\,\tfrac{{\omega }_{i+1+K-m}^{d}}{{\omega }_{i+1+K-m}^{u}})]{\omega }_{i+1}^{u}\\  & = & {B}_{i-1}{\omega }_{i-1}^{d}+{B}_{i+1}{\omega }_{i+1}^{u}.\end{array}$$

Thus, Eq. (26) can be rewritten as ${B}_{i}{\omega }_{i}^{u}+{B}_{i}{\omega }_{i}^{d}-{B}_{i+1}{\omega }_{i+1}^{u}-{B}_{i-1}{\omega }_{i-1}^{d}=0$, which proves that Eq. (24) is the solution of Eq. (11) for arbitrary $i\in \{1,2,\ldots ,K\}$. Therefore, $F={({B}_{1}{B}_{2}\cdots {B}_{K})}^{T}\,$is the solution of Eq. (22). Since the solution is only one-dimensional, the solution of Eq. (22) can be expressed as $F={({f}_{1}{f}_{2}\cdots {f}_{K})}^{T}=Const\cdot {({B}_{1}{B}_{2}\cdots {B}_{K})}^{T}$, where *Const* means a const. Actually, according to the definition of *f*_*i*_, there’ll be no influence on the system if all weight factors increase or reduce the same multiplier at the same time. For simplicity, *Const* is set as one here. Then, according to Eq. (24), density weight can be expressed as ${f}_{i}={B}_{i}=\frac{1}{2K{\omega }_{i}^{d}}(1+{\sum }_{j=1}^{K-1}{\prod }_{m=1}^{j}\,\frac{{\omega }_{i+m}^{u}}{{\omega }_{i+m}^{d}})$ + $\frac{1}{2K{\omega }_{i}^{u}}(1+{\sum }_{j=1}^{K-1}{\prod }_{m=1}^{j}\,\frac{{\omega }_{i+K-m}^{d}}{{\omega }_{i+K-m}^{u}})$ shown in Eq. (12).

### Complex analysis

As for a generalized function $h(s)=ln\frac{{\prod }_{{\rm{i}}=1}^{{\rm{K}}}\,(1+s{f}_{i})}{{s}^{N/L}}$, high order expansion can be obtained: ${\rm{h}}^{\prime} (s)=-\,\frac{{\rm{N}}}{Ls}+{\sum }_{{\rm{i}}=1}^{K}\,\frac{{f}_{i}}{1+s{f}_{i}}$. By employing intermediate value theorem, following equation can be obtained in the real number field:

27 $$\{\begin{array}{l}{\mathrm{lim}}_{{\rm{x}}\to +\infty }\,{\sum }_{{\rm{i}}=1}^{K}\,\frac{x{f}_{i}}{1+x{f}_{i}}=K > \frac{N}{L}\\ {\mathrm{lim}}_{{\rm{x}}\to 0}\,{\sum }_{{\rm{i}}=1}^{K}\,\frac{x{f}_{i}}{1+x{f}_{i}}=0 < \frac{N}{L}\end{array}.$$

Thus, following equation can be satisfied:

28 $${\sum }_{{\rm{i}}=1}^{K}\,\frac{z{{\rm{f}}}_{{\rm{i}}}}{1+z{{\rm{f}}}_{{\rm{i}}}}=\frac{N}{L},$$

where $\,z\in {{\rm{R}}}^{+}$. According to Eq. (28), restriction $-\frac{{\rm{N}}}{{\rm{Lz}}}+{\sum }_{{\rm{i}}=1}^{{\rm{K}}}\,\frac{{{\rm{f}}}_{{\rm{i}}}}{1+{{\rm{zf}}}_{{\rm{i}}}}=0$ can be obtained. Thus, restriction ${\rm{h}}^{\prime} ({\rm{z}})=0$ can be got. As *h*(*s*) is holomorphic in complex number space C\{0}, Taylor expansion can be rewritten as ${\rm{h}}({\rm{s}})={\rm{h}}({\rm{z}})+0.5{\rm{h}}^{\prime\prime} ({\rm{z}}){({\rm{s}}-{\rm{z}})}^{2}+{\rm{O}}({({\rm{s}}-{\rm{z}})}^{3})$. Substituting into Eq. (3), we can obtain the following equation:

29 $$\begin{array}{rcl}{{\rm{Z}}}_{{\rm{L}},{\rm{N}},{\rm{K}}} & = & {\sum }_{{{\rm{M}}}_{1}=0}^{L}\,\ldots \,{\sum }_{{{\rm{M}}}_{{\rm{K}}}=0}^{L}{\prod }_{{\rm{i}}=1}^{{\rm{K}}}\,{{\rm{f}}}_{{\rm{i}}}^{{{\rm{M}}}_{{\rm{i}}}}(\begin{array}{c}L\\ {{\rm{M}}}_{{\rm{i}}}\end{array})\frac{1}{2\pi i}\,\oint \,\frac{{s}^{({{\sum }_{i=1}^{K}{\rm{M}}}_{{\rm{i}}})}}{{s}^{N+1}}ds\\  & = & \frac{1}{2\pi i}\,\oint \,\frac{{Z}_{L,K}(s)}{{s}^{N+1}}ds\\  & = & \frac{1}{2\pi i}\,\oint \,\frac{{{\rm{e}}}^{{\rm{Lh}}({\rm{s}})}}{s}ds\\  & = & \frac{{{\rm{e}}}^{{\rm{Lh}}({\rm{z}})}}{2\pi i}\,\oint \,{{\rm{e}}}^{0.5{{\rm{Lh}}}^{^{\prime\prime} }({\rm{z}}){(s-z)}^{2}+{\rm{o}}({s}^{2})}({z}^{-1}+{\rm{O}}({\rm{s}}-{\rm{z}}))ds\\  & = & \frac{{{\rm{e}}}^{{\rm{Lh}}({\rm{z}})}{(\frac{2}{|{{\rm{h}}}^{^{\prime\prime} }(z)|})}^{0.5}}{2\pi i}\,\oint \,{{\rm{e}}}^{{\rm{L}}{y}^{2}+O({y}^{3})}({z}^{-1}+{\rm{O}}({\rm{y}}))dy\\  & = & \frac{{{\rm{e}}}^{{\rm{Lh}}({\rm{z}})}{(\frac{2}{|{{\rm{h}}}^{^{\prime\prime} }(z)|})}^{0.5}\frac{i}{\sqrt{{\rm{L}}}}}{2\pi i}\,\oint \,{{\rm{e}}}^{-{t}^{2}+{\rm{O}}(\frac{{t}^{3}}{\sqrt{{\rm{L}}}})}({z}^{-1}+{\rm{O}}(t/\sqrt{{\rm{L}}}))dt\\  & = & \sqrt{\frac{1}{2\pi L|{{\rm{h}}}^{^{\prime\prime} }(z)|}}\frac{{{\rm{e}}}^{{\rm{Lh}}({\rm{z}})}}{z},\end{array}$$

where generalized partition function ${Z}_{L,K}(s)$ = $${\sum }_{{{\rm{M}}}_{1}=0}^{L}\,\ldots \,{\sum }_{{{\rm{M}}}_{{\rm{K}}}=0}^{L}{\prod }_{{\rm{i}}=1}^{{\rm{K}}}\,{(s{f}_{i})}^{{{\rm{M}}}_{{\rm{i}}}}(\begin{array}{c}L\\ {{\rm{M}}}_{{\rm{i}}}\end{array})$$ = ${\prod }_{{\rm{i}}=1}^{{\rm{K}}}\,{(1+s{f}_{i})}^{{\rm{L}}}={[{\rm{F}}({\rm{s}})]}^{{\rm{L}}}$. Here, F(s) denotes a generalized function. Thus, ${[{\rm{F}}({\rm{s}})]}^{{\rm{L}}}={{\rm{e}}}^{{\rm{Lh}}({\rm{s}})}{{\rm{s}}}^{{\rm{N}}}$ can be got by introducing variable substitutions ${\rm{s}}={\rm{z}}+{\rm{y}}\sqrt{\frac{2}{|{\rm{h}}^{\prime\prime} (z)|}}$ and ${\rm{t}}=\frac{i\sqrt{{\rm{L}}}}{y}$ into Eq. (29).

Similarly, state number ${Z}_{{\rm{L}},{\rm{N}},{\rm{K}}}^{(i,j)}$ is defined to depict ${{\rm{\tau }}}_{{\rm{i}},{\rm{j}}}=1$. Thus, it can be derived:

30 $$\begin{array}{rcl}{Z}_{{\rm{L}},{\rm{N}},{\rm{K}}}^{(i,j)} & = & \sum _{{{\rm{M}}}_{1}=0}^{{\rm{L}}}\,\cdots \,\sum _{{{\rm{M}}}_{{\rm{i}}}=1}^{{\rm{L}}}\,\cdots \,\sum _{{{\rm{M}}}_{{\rm{K}}}=0}^{{\rm{L}}}{{\rm{f}}}_{{\rm{i}}}^{{{\rm{M}}}_{{\rm{i}}}}(\begin{array}{c}L-1\\ {{\rm{M}}}_{{\rm{i}}}-1\end{array})\,\prod _{{\rm{h}}\ne {\rm{i}}}^{{\rm{K}}}\,{{\rm{f}}}_{{\rm{h}}}^{{{\rm{M}}}_{{\rm{h}}}}(\begin{array}{c}L\\ {{\rm{M}}}_{{\rm{h}}}\end{array})\delta (\sum _{i=1}^{K}\,{{\rm{M}}}_{{\rm{i}}}-N)\\  & = & \frac{1}{2\pi i}\oint \frac{{Z}_{L,K}^{(i,j)}(s)}{{s}^{N+1}}ds\\  & = & \frac{1}{2\pi i}\oint \frac{{f}_{i}{{\rm{e}}}^{{\rm{Lh}}({\rm{s}})}}{1+s{f}_{i}}ds\\  & = & \frac{{{\rm{e}}}^{{\rm{Lh}}({\rm{z}})}}{2\pi i}{f}_{i}\oint {{\rm{e}}}^{{{\rm{Lh}}}^{^{\prime\prime} }({\rm{z}}){(s-z)}^{2}+{\rm{o}}({s}^{2})}(\frac{1}{1+z{f}_{i}}+{\rm{O}}({\rm{s}}-{\rm{z}}))ds\\  & = & \tfrac{{{\rm{e}}}^{{\rm{Lh}}({\rm{z}})}{f}_{i}{(\tfrac{2}{|{{\rm{h}}}^{^{\prime\prime} }({\rm{z}})|})}^{0.5}}{2\pi i}\oint {{\rm{e}}}^{{\rm{L}}{y}^{2}+{\rm{O}}({y}^{3})}(\frac{1}{1+z{f}_{i}}+{\rm{O}}({\rm{y}}))dy\\  & = & \tfrac{{{\rm{e}}}^{{\rm{Lh}}({\rm{z}})}{f}_{i}{(\tfrac{2}{|{{\rm{h}}}^{^{\prime\prime} }({\rm{z}})|})}^{0.5}\tfrac{i}{\sqrt{{\rm{L}}}}}{2\pi i}\oint {{\rm{e}}}^{-{t}^{2}+{\rm{O}}(\frac{{t}^{3}}{\sqrt{{\rm{L}}}})}(\frac{1}{1+z{f}_{i}}+{\rm{O}}(t/\sqrt{{\rm{L}}}))dt\\  & = & \sqrt{\frac{1}{2\pi L|{{\rm{h}}}^{^{\prime\prime} }({\rm{z}})|}}\frac{{{\rm{e}}}^{{\rm{Lh}}({\rm{z}})}{f}_{i}}{1+z{f}_{i}},\end{array}$$

where ${Z}_{L,K}^{(i,j)}(s)$ = $${\sum }_{{{\rm{M}}}_{1}=0}^{{\rm{L}}}\,\cdots \,{\sum }_{{{\rm{M}}}_{{\rm{i}}}=1}^{{\rm{L}}}\,\cdots \,{\sum }_{{{\rm{M}}}_{{\rm{K}}}=0}^{{\rm{L}}}\,{{\rm{f}}}_{{\rm{i}}}^{{{\rm{M}}}_{{\rm{i}}}}{s}^{{{\rm{M}}}_{{\rm{i}}}}(\begin{array}{c}L-1\\ {{\rm{M}}}_{{\rm{i}}}-1\end{array})\,{\prod }_{{\rm{h}}\ne {\rm{i}}}^{{\rm{K}}}\,{{\rm{f}}}_{{\rm{h}}}^{{{\rm{M}}}_{{\rm{h}}}}(\begin{array}{c}L\\ {{\rm{M}}}_{{\rm{h}}}\end{array}){s}^{{{\rm{M}}}_{{\rm{h}}}}$$ = $\frac{s{f}_{i}}{1+s{f}_{i}}{[{\rm{F}}({\rm{s}})]}^{{\rm{L}}}$ is satisfied. Thus, based on Eqs (12), (29) and (30), the local density ${\rho }_{i}$ can be derived:

31 $${\rho }_{i}=\tfrac{{Z}_{{\rm{L}},{\rm{N}},{\rm{K}}}^{(i,j)}}{{Z}_{L,N,K}}=\tfrac{\sqrt{\tfrac{1}{2\pi L|{{\rm{h}}}^{^{\prime\prime} }({\rm{z}})|}}\tfrac{{{\rm{e}}}^{{\rm{Lh}}({\rm{z}})}{f}_{i}}{1+z{f}_{i}}}{\sqrt{\tfrac{1}{2\pi L|{{\rm{h}}}^{^{\prime\prime} }({\rm{z}})|}}\tfrac{{{\rm{e}}}^{{\rm{Lh}}({\rm{z}})}}{z}}=\tfrac{{\rm{z}}(\tfrac{1}{2K{\omega }_{i}^{d}}(1+{\sum }_{j=1}^{K-1}\,{\prod }_{m=1}^{j}\,\tfrac{{\omega }_{i+m}^{u}}{{\omega }_{i+m}^{d}})+\tfrac{1}{2K{\omega }_{i}^{u}}(1+{\sum }_{j=1}^{K-1}\,{\prod }_{m=1}^{j}\,\tfrac{{\omega }_{i+K-m}^{d}}{{\omega }_{i+K-m}^{u}}))}{1+{\rm{z}}(\tfrac{1}{2K{\omega }_{i}^{d}}(1+{\sum }_{j=1}^{K-1}\,{\prod }_{m=1}^{j}\,\tfrac{{\omega }_{i+m}^{u}}{{\omega }_{i+m}^{d}})+\tfrac{1}{2K{\omega }_{i}^{u}}(1+{\sum }_{j=1}^{K-1}\,{\prod }_{m=1}^{j}\,\tfrac{{\omega }_{i+K-m}^{d}}{{\omega }_{i+K-m}^{u}}))}$$

shown in Eq. (14).

Additionally, another important characteristic parameter, the local current *J*_*i*_, also needs to be investigated. As update rule, hopping can occur when ${{\rm{\tau }}}_{{\rm{i}},{\rm{j}}}=1$ and ${{\rm{\tau }}}_{{\rm{i}},{\rm{j}}+1}=0$. Similarly, partition functions can be obtained:

32 $${Z}_{{\rm{L}},{\rm{N}},{\rm{K}}}^{(i,j)\to (i,j+1)}=\sqrt{\frac{1}{2\pi L|{{\rm{h}}}^{^{\prime\prime} }({\rm{z}})|}}\frac{{{\rm{e}}}^{{\rm{Lh}}({\rm{z}})}{f}_{i}}{{(1+z{f}_{i})}^{2}}.$$

Thus, based on Eqs (29) and (32), *J*_*i*_ can be obtained:

33 $$\begin{array}{rcl}{J}_{i} & = & {p}_{i}\tfrac{{Z}_{{\rm{L}},{\rm{N}},{\rm{K}}}^{(i,j)\to (i,j+1)}}{{Z}_{L,N,K}}\\  & = & {p}_{i}\tfrac{\sqrt{\tfrac{1}{2\pi L|{{\rm{h}}}^{^{\prime\prime} }({\rm{z}})|}}\tfrac{{{\rm{e}}}^{{\rm{Lh}}({\rm{z}})}{f}_{i}}{{(1+z{f}_{i})}^{2}}}{\sqrt{\tfrac{1}{2\pi L|{{\rm{h}}}^{^{\prime\prime} }({\rm{z}})|}}\tfrac{{{\rm{e}}}^{{\rm{Lh}}({\rm{z}})}}{z}}\\  & = & {p}_{i}\tfrac{z(\tfrac{1}{2K{\omega }_{i}^{d}}(1+{\sum }_{j=1}^{K-1}{\prod }_{m=1}^{j}\tfrac{{\omega }_{i+m}^{u}}{{\omega }_{i+m}^{d}})+\tfrac{1}{2K{\omega }_{i}^{u}}(1+{\sum }_{j=1}^{K-1}{\prod }_{m=1}^{j}\tfrac{{\omega }_{i+K-m}^{d}}{{\omega }_{i+K-m}^{u}}))}{{(1+z(\tfrac{1}{2K{\omega }_{i}^{d}}(1+{\sum }_{j=1}^{K-1}{\prod }_{m=1}^{j}\tfrac{{\omega }_{i+m}^{u}}{{\omega }_{i+m}^{d}})+\tfrac{1}{2K{\omega }_{i}^{u}}(1+{\sum }_{j=1}^{K-1}{\prod }_{m=1}^{j}\tfrac{{\omega }_{i+K-m}^{d}}{{\omega }_{i+K-m}^{u}})))}^{2}}\end{array}$$

displayed in Eq. (15). Moreover, based on Eq. (31), expectation $\langle {{\rm{n}}}_{{\rm{i}}}\rangle$ of number of particles in subsystem can be obtained:

34 $$\begin{array}{rcl}\langle {{\rm{n}}}_{{\rm{i}}}\rangle  & = & {\rm{E}}\langle {{\rm{\tau }}}_{{\rm{i}}}\rangle \\  & = & {\sum }_{{\rm{j}}=1}^{{\rm{L}}}\,{\rm{E}}\langle {{\rm{\tau }}}_{{\rm{i}},{\rm{j}}}\rangle \\  & = & \,{\sum }_{{\rm{j}}=1}^{{\rm{L}}}\,{\rm{P}}({{\rm{\tau }}}_{{\rm{i}},{\rm{j}}}=1)\\  & = & L{\rho }_{i}\\  & = & L\tfrac{{\rm{z}}(\tfrac{1}{2K{\omega }_{i}^{d}}(1+{\sum }_{j=1}^{K-1}\,{\prod }_{m=1}^{j}\,\tfrac{{\omega }_{i+m}^{u}}{{\omega }_{i+m}^{d}})+\tfrac{1}{2K{\omega }_{i}^{u}}(1+{\sum }_{j=1}^{K-1}\,{\prod }_{m=1}^{j}\,\tfrac{{\omega }_{i+K-m}^{d}}{{\omega }_{i+K-m}^{u}}))}{1+{\rm{z}}(\tfrac{1}{2K{\omega }_{i}^{d}}(1+{\sum }_{j=1}^{K-1}\,{\prod }_{m=1}^{j}\,\tfrac{{\omega }_{i+m}^{u}}{{\omega }_{i+m}^{d}})+\tfrac{1}{2K{\omega }_{i}^{u}}(1+{\sum }_{j=1}^{K-1}\,{\prod }_{m=1}^{j}\,\tfrac{{\omega }_{i+K-m}^{d}}{{\omega }_{i+K-m}^{u}}))}.\end{array}$$

Similarly, based on Eq. (31), variance ${\rm{D}}\langle {{\rm{n}}}_{{\rm{i}}}\rangle$ of number of particles in subsystem can be derived:

35 $$\begin{array}{rcl}{\rm{D}}\langle {{\rm{n}}}_{{\rm{i}}}\rangle  & = & {\sum }_{{\rm{j}}=1}^{{\rm{L}}}\,{\rm{D}}({{\rm{\tau }}}_{{\rm{i}},{\rm{j}}})\\  & = & {\sum }_{{\rm{j}}=1}^{{\rm{L}}}\,({\rm{E}}({{{\rm{\tau }}}_{{\rm{i}},{\rm{j}}}}^{2})-{({\rm{E}}({{\rm{\tau }}}_{{\rm{i}},{\rm{j}}}))}^{2})\\  & = & {\sum }_{{\rm{j}}=1}^{{\rm{L}}}\,({\rho }_{i}\,-{{\rho }_{i}}^{2})\\  & = & \tfrac{{\rm{zL}}[\tfrac{1}{2K{\omega }_{i}^{d}}(1+{\sum }_{j=1}^{K-1}\,{\prod }_{m=1}^{j}\,\tfrac{{\omega }_{i+m}^{u}}{{\omega }_{i+m}^{d}})+\tfrac{1}{2K{\omega }_{i}^{u}}(1+{\sum }_{j=1}^{K-1}\,{\prod }_{m=1}^{j}\,\tfrac{{\omega }_{i+K-m}^{d}}{{\omega }_{i+K-m}^{u}})]}{{\{1+{\rm{z}}[\tfrac{1}{2K{\omega }_{i}^{d}}(1+{\sum }_{j=1}^{K-1}{\prod }_{m=1}^{j}\tfrac{{\omega }_{i+m}^{u}}{{\omega }_{i+m}^{d}})+\tfrac{1}{2K{\omega }_{i}^{u}}(1+{\sum }_{j=1}^{K-1}{\prod }_{m=1}^{j}\tfrac{{\omega }_{i+K-m}^{d}}{{\omega }_{i+K-m}^{u}})]\}}^{2}}.\end{array}$$

Finally, effects of fully heterogeneous interactions on global transport are discussed. Firstly, total current J_total_ can be expressed:

36 $${{\rm{J}}}_{{\rm{total}}}={\sum }_{{\rm{i}}=1}^{{\rm{K}}}\,({{\rm{p}}}_{{\rm{i}}}\tfrac{{\rm{z}}\,[\tfrac{1}{2K{\omega }_{i}^{d}}(1+{\sum }_{j=1}^{K-1}\,{\prod }_{m=1}^{j}\,\tfrac{{\omega }_{i+m}^{u}}{{\omega }_{i+m}^{d}})+\tfrac{1}{2K{\omega }_{i}^{u}}(1+{\sum }_{j=1}^{K-1}\,{\prod }_{m=1}^{j}\,\tfrac{{\omega }_{i+K-m}^{d}}{{\omega }_{i+K-m}^{u}})]}{{\{1+{\rm{z}}[\tfrac{1}{2K{\omega }_{i}^{d}}(1+{\sum }_{j=1}^{K-1}{\prod }_{m=1}^{j}\tfrac{{\omega }_{i+m}^{u}}{{\omega }_{i+m}^{d}})+\tfrac{1}{2K{\omega }_{i}^{u}}(1+{\sum }_{j=1}^{K-1}{\prod }_{m=1}^{j}\tfrac{{\omega }_{i+K-m}^{d}}{{\omega }_{i+K-m}^{u}})]\}}^{2}}).$$

Then, generalized function below is introduced to obtain the maximum value of J_total_:

37 $$\begin{array}{l}{\rm{F}}({\omega }_{1}^{d},{\omega }_{1}^{u},\ldots ,{\omega }_{K}^{d},{\omega }_{K}^{u},{\rm{\lambda }})\\ \begin{array}{rcl} & = & {\sum }_{{\rm{i}}=1}^{{\rm{K}}}\,({{\rm{p}}}_{{\rm{i}}}\tfrac{{\rm{z}}\,[\tfrac{1}{2K{\omega }_{i}^{d}}(1+{\sum }_{j=1}^{K-1}\,{\prod }_{m=1}^{j}\,\tfrac{{\omega }_{i+m}^{u}}{{\omega }_{i+m}^{d}})+\tfrac{1}{2K{\omega }_{i}^{u}}(1+{\sum }_{j=1}^{K-1}\,{\prod }_{m=1}^{j}\,\tfrac{{\omega }_{i+K-m}^{d}}{{\omega }_{i+K-m}^{u}})]}{{\{1+{\rm{z}}[\tfrac{1}{2K{\omega }_{i}^{d}}(1+{\sum }_{j=1}^{K-1}{\prod }_{m=1}^{j}\tfrac{{\omega }_{i+m}^{u}}{{\omega }_{i+m}^{d}})+\tfrac{1}{2K{\omega }_{i}^{u}}(1+{\sum }_{j=1}^{K-1}{\prod }_{m=1}^{j}\tfrac{{\omega }_{i+K-m}^{d}}{{\omega }_{i+K-m}^{u}})]\}}^{2}})\\  &  & +\,{\rm{\lambda }}\,{\sum }_{{\rm{i}}=1}^{{\rm{K}}}\,(\tfrac{{\rm{z}}\,[\tfrac{1}{2K{\omega }_{i}^{d}}(1+{\sum }_{j=1}^{K-1}\,{\prod }_{m=1}^{j}\,\tfrac{{\omega }_{i+m}^{u}}{{\omega }_{i+m}^{d}})+\tfrac{1}{2K{\omega }_{i}^{u}}(1+{\sum }_{j=1}^{K-1}\,{\prod }_{m=1}^{j}\,\tfrac{{\omega }_{i+K-m}^{d}}{{\omega }_{i+K-m}^{u}})]}{1+{\rm{z}}\,[\tfrac{1}{2K{\omega }_{i}^{d}}(1+{\sum }_{j=1}^{K-1}\,{\prod }_{m=1}^{j}\,\tfrac{{\omega }_{i+m}^{u}}{{\omega }_{i+m}^{d}})+\tfrac{1}{2K{\omega }_{i}^{u}}(1+{\sum }_{j=1}^{K-1}\,{\prod }_{m=1}^{j}\,\tfrac{{\omega }_{i+K-m}^{d}}{{\omega }_{i+K-m}^{u}})]}),\end{array}\end{array}$$

where λ means Lagrange multiplier. When J_total_ reaches the maximum, constraints $\frac{\partial F}{\partial {\omega }_{i}^{u}}=0$ and $\frac{\partial F}{\partial {\omega }_{i}^{d}}=0$ are satisfied for arbitrary ${\rm{i}}\in \{1\ldots K\}$. Thus, by combining Eqs (36) and (37), following equation can be obtained:

38 $$\tfrac{{\rm{z}}\,[\tfrac{1}{2K{\omega }_{i}^{d}}(1+{\sum }_{j=1}^{K-1}\,{\prod }_{m=1}^{j}\,\tfrac{{\omega }_{i+m}^{u}}{{\omega }_{i+m}^{d}})+\tfrac{1}{2K{\omega }_{i}^{u}}(1+{\sum }_{j=1}^{K-1}\,{\prod }_{m=1}^{j}\,\tfrac{{\omega }_{i+K-m}^{d}}{{\omega }_{i+K-m}^{u}})]}{1+{\rm{z}}\,[\tfrac{1}{2K{\omega }_{i}^{d}}(1+{\sum }_{j=1}^{K-1}\,{\prod }_{m=1}^{j}\,\tfrac{{\omega }_{i+m}^{u}}{{\omega }_{i+m}^{d}})+\tfrac{1}{2K{\omega }_{i}^{u}}(1+{\sum }_{j=1}^{K-1}\,{\prod }_{m=1}^{j}\,\tfrac{{\omega }_{i+K-m}^{d}}{{\omega }_{i+K-m}^{u}})]}=0.5(1-\frac{{\rm{\lambda }}}{{p}_{i}}).$$

As ${\sum }_{{\rm{i}}=1}^{{\rm{K}}}\,(\tfrac{{\rm{z}}\,[\tfrac{1}{2K{\omega }_{i}^{d}}(1+{\sum }_{j=1}^{K-1}\,{\prod }_{m=1}^{j}\,\tfrac{{\omega }_{i+m}^{u}}{{\omega }_{i+m}^{d}})+\tfrac{1}{2K{\omega }_{i}^{u}}(1+{\sum }_{j=1}^{K-1}\,{\prod }_{m=1}^{j}\,\tfrac{{\omega }_{i+K-m}^{d}}{{\omega }_{i+K-m}^{u}})]}{1+{\rm{z}}\,[\tfrac{1}{2K{\omega }_{i}^{d}}(1+{\sum }_{j=1}^{K-1}\,{\prod }_{m=1}^{j}\,\tfrac{{\omega }_{i+m}^{u}}{{\omega }_{i+m}^{d}})+\tfrac{1}{2K{\omega }_{i}^{u}}(1+{\sum }_{j=1}^{K-1}\,{\prod }_{m=1}^{j}\,\tfrac{{\omega }_{i+K-m}^{d}}{{\omega }_{i+K-m}^{u}})]})=\frac{{\rm{N}}}{{\rm{L}}}$, following equation can be derived by combining Eq. (38):

39 $${\rm{\lambda }}=\frac{0.5\,{\rm{KL}}-{\rm{N}}}{{\rm{L}}\,{\sum }_{{\rm{i}}=1}^{{\rm{K}}}\,\frac{1}{{p}_{{\rm{i}}}}}.$$

Thus, the maximum value J_max_ of total current can be obtained:

40 $${{\rm{J}}}_{{\rm{\max }}}={\sum }_{{\rm{i}}=1}^{{\rm{K}}}\,(0.25({{\rm{p}}}_{{\rm{i}}}\,{\sum }_{{\rm{i}}=1}^{{\rm{K}}}\,\frac{1}{{{\rm{p}}}_{{\rm{i}}}}-0.5{\rm{K}}+\frac{{\rm{N}}}{{\rm{L}}})({{\rm{p}}}_{{\rm{i}}}\,{\sum }_{{\rm{i}}=1}^{{\rm{K}}}\,\frac{1}{{{\rm{p}}}_{{\rm{i}}}}+0.5{\rm{K}}-\frac{{\rm{N}}}{{\rm{L}}})/({{\rm{p}}}_{{\rm{i}}}{({\sum }_{{\rm{i}}=1}^{{\rm{K}}}\frac{1}{{{\rm{p}}}_{{\rm{i}}}})}^{2}))$$

shown in Eq. (19). Besides, extreme condition can also be got:

41 $$\tfrac{{\rm{z}}\,[\tfrac{1}{2K{\omega }_{i}^{d}}(1+{\sum }_{j=1}^{K-1}\,{\prod }_{m=1}^{j}\,\tfrac{{\omega }_{i+m}^{u}}{{\omega }_{i+m}^{d}})+\tfrac{1}{2K{\omega }_{i}^{u}}(1+{\sum }_{j=1}^{K-1}\,{\prod }_{m=1}^{j}\,\tfrac{{\omega }_{i+K-m}^{d}}{{\omega }_{i+K-m}^{u}})]}{1+{\rm{z}}\,[\tfrac{1}{2K{\omega }_{i}^{d}}(1+{\sum }_{j=1}^{K-1}\,{\prod }_{m=1}^{j}\,\tfrac{{\omega }_{i+m}^{u}}{{\omega }_{i+m}^{d}})+\tfrac{1}{2K{\omega }_{i}^{u}}(1+{\sum }_{j=1}^{K-1}\,{\prod }_{m=1}^{j}\,\tfrac{{\omega }_{i+K-m}^{d}}{{\omega }_{i+K-m}^{u}})]}=\tfrac{0.5({p}_{i}{\sum }_{i=1}^{K}\,\tfrac{1}{{p}_{i}}-0.5K+\tfrac{N}{L})}{{p}_{i}\,{\sum }_{i=1}^{K}\,\tfrac{1}{{p}_{i}}},$$

which means that J_max_ achieves when interaction rates ${\omega }_{i}^{u}$ and ${\omega }_{i}^{d}$ satisfy the constraint Eq. (41).
